# Exogenous H_2_S initiating Nrf2/GPx4/GSH pathway through promoting Syvn1-Keap1 interaction in diabetic hearts

**DOI:** 10.1038/s41420-023-01690-w

**Published:** 2023-10-24

**Authors:** Mengyi Wang, Jingyuan Tang, Shiwu Zhang, Kemiao Pang, Yajun Zhao, Ning Liu, Jiayi Huang, Jiaxin Kang, Shiyun Dong, Hongxia Li, Zhen Tian, Binhong Duan, Fanghao Lu, Weihua Zhang

**Affiliations:** 1 Department of Pathophysiology, Harbin Medical University, 150081 Harbin, China; 2 Department of Endocrinology, Heilongjiang Provincial Hospital, 150036 Harbin, China

**Keywords:** Ubiquitylation, Heart failure, Apoptosis

## Abstract

Excessive ROS accumulation contributes to cardiac injury in type 2 diabetes mellitus. Hydrogen sulfide (H_2_S) is a vital endogenous gasotransmitter to alleviate cardiac damage in diabetic cardiomyopathy (DCM). However, the underlying mechanisms remain unclear. In this study, we investigated the effects of NaHS administration in db/db mice via intraperitoneal injection for 20 weeks and the treatment of high glucose (HG), palmitate (PA) and NaHS in HL-1 cardiomyocytes for 48 h, respectively. H_2_S levels were decreased in hearts of db/db mice and HL-1 cardiomyocytes exposed to HG and PA, which were restored by NaHS. Exogenous H_2_S activated the nuclear factor erythroid 2-related factor 2 (Nrf2)/glutathione peroxidase 4 (GPx4)/glutathione (GSH) pathway, suppressed ferroptosis and mitigated mitochondrial apoptosis in db/db mice. However, these effects were abrogated after Nrf2 knockdown. NaHS treatment elevated the ubiquitination level of Kelch-like ECH-associated protein (Keap1) by preserving its E3 ligase synoviolin (Syvn1), resulting in Nrf2 nuclear translocation. H_2_S facilitated the sulfhydration of Syvn1-cys115 site, a post-translational modification. Transfecting Syvn1 C115A in cardiomyocytes exposed to HG and PA partially attenuated the effects of NaHS on Nrf2 and cell death. Our findings suggest that exogenous H_2_S regulates Nrf2/GPx4/GSH pathway by promoting the Syvn1-Keap1 interaction to reduce ferroptosis and mitochondrial apoptosis in DCM.

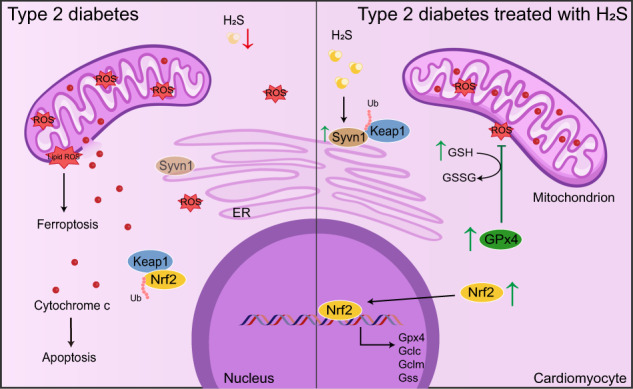

## Introduction

According to the International Diabetes Federation Diabetes Atlas report, the global prevalence of diabetes in adults exceeds 500 million, and this number is projected to reach 700 million by 2045. Consequently, there is a growing epidemic of diabetes-induced heart failure, commonly known as diabetic cardiomyopathy (DCM) [1]. DCM is characterized by impaired cardiac contraction in the absence of coronary artery disease, hypertension and valvular heart disease [2]. Multiple mechanisms contribute to the development of DCM, including hyperglycemia along with increased fatty acids and cytokines, Advanced Glycation End-products (AGE) formation and increased production of ROS [3]. Emerging evidence indicates that excessive ROS production disrupts the antioxidant system, leading to cardiac tissue injury [4].

Nuclear factor erythroid 2-related factor 2 (Nrf2) is a crucial transcription factor involved in the antioxidant system. The mammalian Nrf2 gene encodes a 605-amino-acid protein with seven functional domains, known as Nrf2-ECH homology (Neh) 1–7. The Neh1 domain consists of a basic leucine zipper (bZIP) motif responsible for DNA binding and transcriptional activation. The Neh2 domain contains the binding sites for Kelch-like ECH-associated protein (Keap1). Under physiological conditions, Nrf2 is constitutively degraded through the ubiquitin–proteasome pathway with Keap1 serving as its binding partner to facilitate this process [5, 6]. However, under oxidative stress, Nrf2 stimulates the expression of several redox genes, such as glutathione synthetase (*Gss*), glutathione peroxidase 4 (*Gpx4*), glutamate-cysteine ligase catalytic subunit (*Gclc*), *Slc7a11*, superoxide dismutase (*Sod*) and catalase (*Cat*) [7, 8]. The Nrf2-mediated antioxidant response plays an important role in various cardiac disorders, including ischemic heart disease and dilated cardiomyopathy. Notably, GPx4, a downstream target of Nrf2, is localized in mitochondria and cytoplasm, where it scavenges lipid peroxide (lipid ROS) and suppresses ferroptosis. Lipid ROS is generated by peroxidation of polyunsaturated fatty acids (PUFAs) in membrane components, thereby inducing ferroptosis [9].

Hydrogen sulfide (H_2_S) is a crucial physiological gaseous molecule that regulates cardiovascular homeostasis [10]. It is generated by cystathionine γ-lyase (CSE), cystathionine β-synthase (CBS) and 3-mercaptopyruvate sulfurtransferase (3-MST) [11]. CSE is extensively expressed in the cardiovascular system. Previous studies have demonstrated that H_2_S takes part in several protective actions, including ATP synthesis, mitochondrial respiration and the reduction of basal levels of cardiac ROS. Additionally, H_2_S also modifies the free thiol groups of target proteins through cysteine persulfides, thereby altering the structure and function of these proteins. However, whether alterations in H_2_S production contribute to DCM has yet to be investigated. In the current study, we aimed to test the hypothesis that H_2_S preserves the protein level of Nrf2 by regulating Keap1 ubiquitination. This mechanism helps to maintain the expression of antioxidant proteins and mitochondrial function, ultimately inhibiting cardiomyocyte death under hyperglycemic and hyperlipidemic conditions.

## Results

### Endogenous H_2_S level is decreased in type 2 diabetic models

To assess the effects of NaHS treatment, we first examined the basic parameters of mice. We found that db/db mice exhibited significantly higher body weight and blood glucose levels compared to WT mice (Supplementary Fig. 1A). Additionally, db/db mice showed impaired glucose tolerance (Supplementary Fig. 1B). These data confirm that db/db mice are typical animal models of type 2 diabetes.

**Fig. 1:**
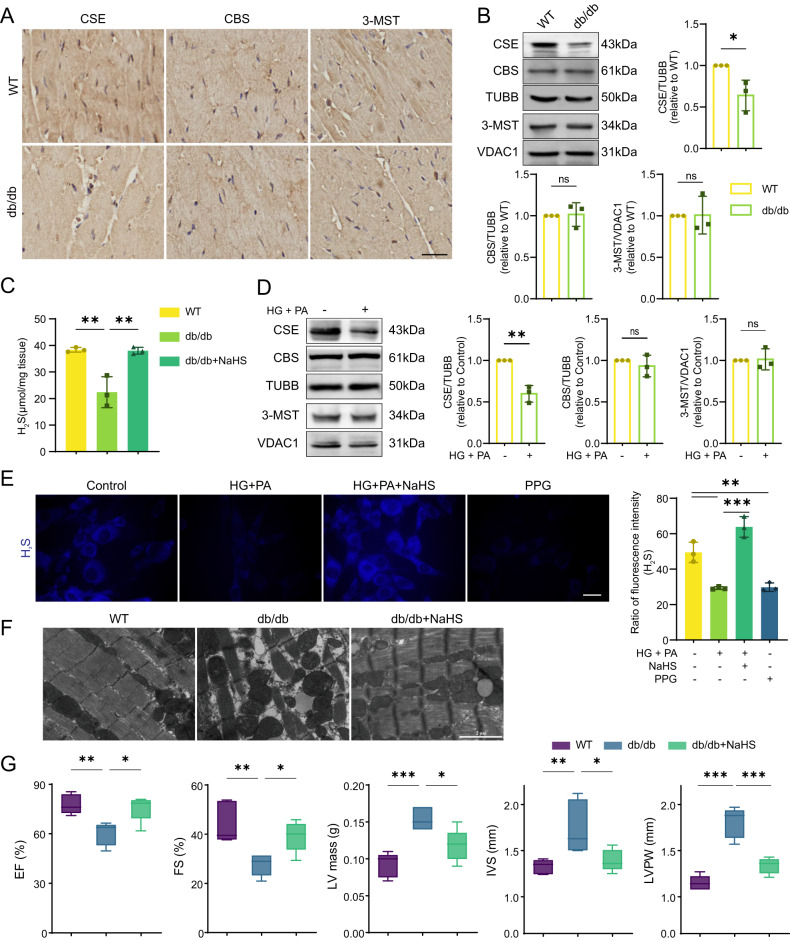
Endogenous H_2_S level is decreased in type 2 diabetic models. **A** Immunohistochemistry (IHC) staining of CSE, CBS and 3-MST in cardiac tissues. Scale bar: 25 μm. **B** Western blot (WB) showing the expression of CSE, CBS and 3-MST in cardiac tissues, *n* = 3. **C** H_2_S concentration in cardiac tissues, *n* = 3. **D** WB showing CSE, CBS and 3-MST levels in HL-1 cardiomyocytes, *n* = 3. **E** H_2_S content in HL-1 cardiomyocytes stained by fluorescence probe C-7Az, *n* = 3. Scale bar: 50 μm. **F** Transmission electron microscope (TEM) images of cardiac tissues. Scale bar: 2 μm. **G** Heart functions measured by heart echocardiography, *n* = 5. EF ejection fraction, FS fractional shortening, LV mass left ventricular mass, IVS interventricular septal, FVPW left ventricular end-diastolic posterior wall. All quantitative data are presented as mean ± SD from independent experiments. *P < 0.05, **P < 0.01, ***P < 0.001 by unpaired *t* test or ordinary one-way ANOVA.

H_2_S has been reported to play multiple physiological roles in cardiovascular homeostasis, including antioxidation and anti-inflammation [12, 13]. We evaluated the expression of CSE, CBS and 3-MST, which are major enzymes to generate endogenous H_2_S. We observed a decrease in expression of CSE, however, the protein levels of CBS and 3-MST showed no difference between WT and db/db mice via IHC and western blot analysis (Fig. 1A, B). Consistent with CSE downregulation, cardiac H_2_S generation was reduced by approximately 33% in db/db mice (Fig. 1C).

To further elucidate the CSE/H_2_S changes under hyperglycemic and hyperlipidemic conditions, we employed HL-1 cardiomyocytes with the treatment of glucose (HG) and palmitate (PA) as a cellular model. Similar patterns of CSE, CBS and 3-MST expression were observed in the cultured HL-1 cardiomyocytes and db/db mice (Fig. 1D). H_2_S levels in HL-1 cardiomyocytes were detected using the H_2_S probe, C-7Az. The H_2_S levels were significantly decreased in the HG + PA groups, but they were obviously restored by NaHS treatment. DL-propargylglycine (PPG), a CSE inhibitor, also reduced H_2_S production (Fig. 1E).

To further explore whether exogenous H_2_S alters cardiac structure in type 2 diabetes, we performed transmission electron microscopy (TEM) analysis on left ventricular tissues from mice. TEM images revealed that the mitochondria in WT mice exhibited a regular arrangement in close proximity to sarcomeric myofibrils, with no significant differences in size. However, in db/db mice, the myocardial fiber structure appeared disarrayed, and the mitochondria became swelling, wrinkling, and rupture, accompanied by loss of cristae integrity (Fig. 1F). NaHS treatment induced mitochondrial size uniformity and prevented mitochondrial fragmentation and cristae disorganization (Fig. 1F). These results verify a positive effect of exogenous H_2_S on maintaining mitochondrial morphology.

Moreover, the ejection fraction (EF)%, fractional shortening (FS)%, left ventricular mass (LV mass), interventricular septal (IVS) and left ventricular end-diastolic posterior wall (LVPW) were analyzed using echocardiography. The values of EF% and FS% were obviously decreased in db/db mice and partially recovered by NaHS treatment. Additionally, LV mass, IVS and LVPW in db/db mice were increased compared with WT and NaHS-treated db/db mice, indicating that exogenous H_2_S improves cardiac contraction of db/db mice effectively (Fig. 1G). These results demonstrate that the decrease of endogenous H_2_S in the hearts of db/db mice is sufficient to cause cardiac dysfunction.

### Exogenous H_2_S improves the redox state of cardiomyocytes

**Fig. 2:**
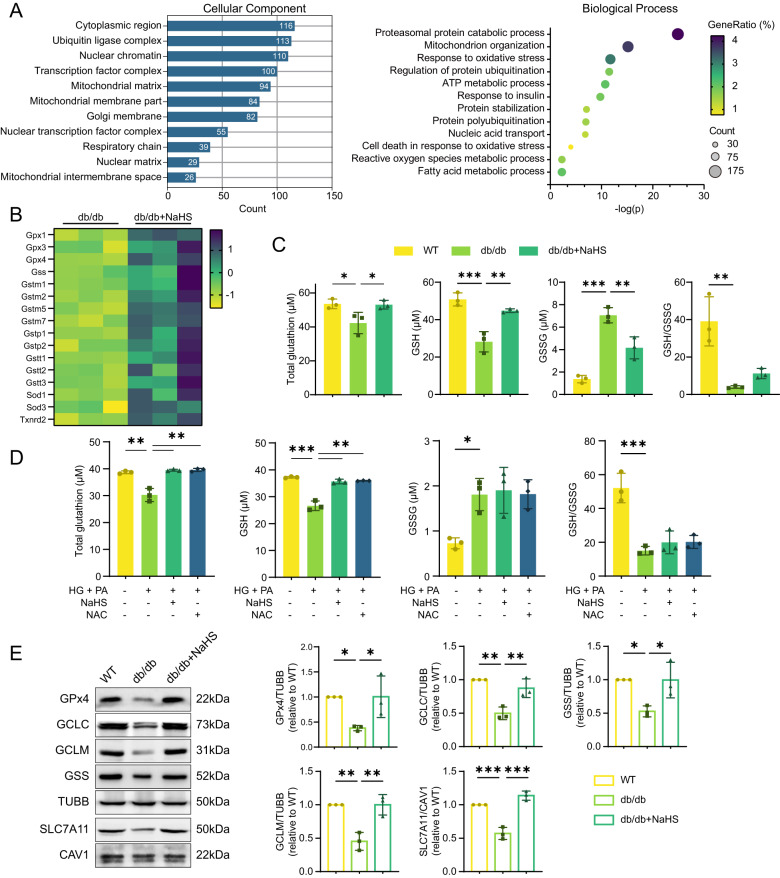
Exogenous H_2_S promotes the GPx4/GSH signaling in db/db mouse myocardium. **A** Gene ontology (GO) classification of differentially expressed genes (DEGs) between hearts of db/db mice and NaHS-treated db/db mice by mRNA sequencing analysis, *n* = 3. **B** Genes associated with antioxidant system among DEGs between db/db and NaHS-treated db/db mice, *n* = 3. **C**, **D** Total glutathione, reduced glutathione (GSH) and oxidative glutathione (GSSG) concentration in (**C**) cardiac tissues, *n* = 3 and in (**D**) HL-1 cardiomyocytes, *n* = 3. **E** WB showing expression of GPx4, GCLC, GCLM, GSS and SLC7A11 in cardiac tissues, *n* = 3. All quantitative data are presented as mean ± SD from independent experiments. *P < 0.05, **P < 0.01, ***P < 0.001 by ordinary one-way ANOVA.

To clarify the molecular mechanisms underlying the protective effects of H_2_S in DCM, we performed cardiac transcriptome analysis in db/db mice and NaHS-treated db/db mice. Gene ontology (GO) classification of differentially expressed genes (DEGs) between the two groups were shown in Fig. 2A. Among these DEGs, 113 genes were identified in ubiquitin ligase complex, 100 in transcription factor complex and 29 in nuclear matrix. The annotation of biological processes indicated that DEGs were enriched in processes including proteasomal proteins, response to oxidative stress and regulation of protein ubiquitination (Fig. 2A). Furthermore, several genes involved in the antioxidant system were upregulated after NaHS treatment (Fig. 2B).

Based on the cardiac transcriptome analysis, we examined the major components of the antioxidant system, GPx4 and reduced glutathione (GSH). In db/db mice, total glutathione and GSH levels were decreased, whereas the oxidized GSSG levels were significantly increased, resulting in a diminished GSH/GSSG ratio (Fig. 2C). Similar patterns of GSH and GSSG concentrations were observed in HL-1 cardiomyocytes treated with HG and PA. Treatment with N-Acetyl-l-cysteine (NAC), an antioxidant and precursor for GSH synthesis, restored the cellular levels of GSH (Fig. 2D). These findings indicate that hyperglycemia and hyperlipidemia disrupt cardiac antioxidant balance and trigger oxidative stress, which can be reversed by exogenous H_2_S.

Next, we evaluated the expression of GPx4 and enzymes involved in GSH synthesis, including GCLC, GCLM and GSS. The protein levels of GPx4, GCLC, GCLM, GSS and the cystine transporter SLC7A11 were dramatically decreased in db/db mice, while NaHS treatment restored their expression (Fig. 2E). Consistent with protein expression, mRNA levels of *Gpx4*, *Gclc*, *Gclm* and *Gss* were also reduced in db/db mice and restored by NaHS treatment (Supplementary Fig. 2A). Similar patterns of protein levels were obtained in HL-1 cardiomyocytes when CSE was inhibited (Supplementary Fig. 2B).

Oxidative stress is related to overwhelming ROS production, with mitochondria being the main source of ROS through the electron transport chain [14, 15]. GPx4 and GSH play crucial roles in scavenging ROS [9]. Therefore, we used fluorescent probes to detect ROS levels in cytoplasm and mitochondria of HL-1 cardiomyocytes. Our results showed that ROS levels were apparently increased in the HG + PA and PPG groups compared to the control and NaHS groups (Supplementary Fig. 3), revealing an imbalance in redox homeostasis in cardiomyocyte mitochondria under hyperglycemic and hyperlipidemic conditions.

### Exogenous H_2_S protects cardiomyocytes from ferroptosis and mitochondrial damage

**Fig. 3:**
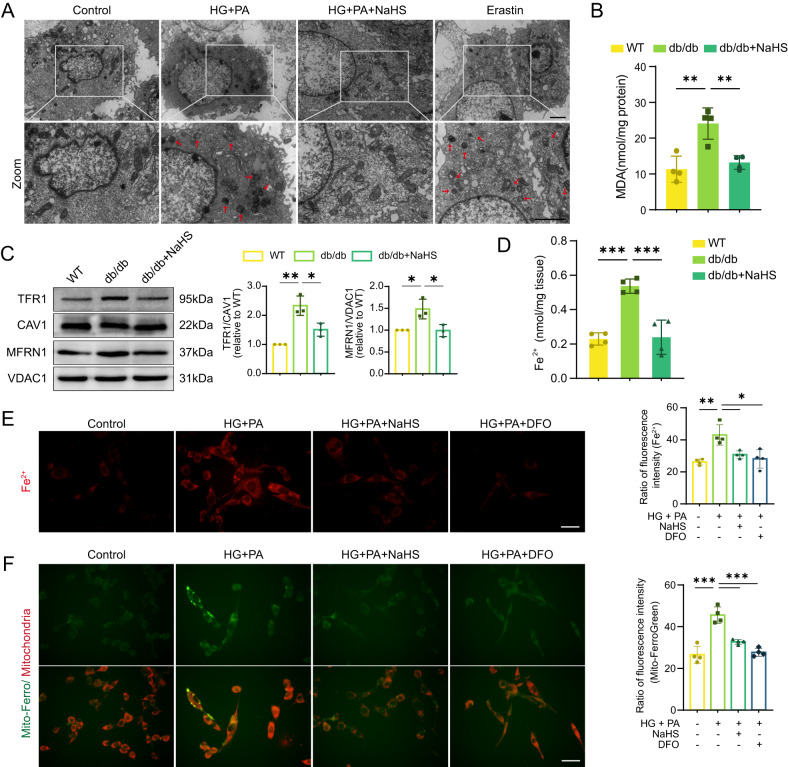
Exogenous H_2_S inhibits ferroptosis in cardiomyocytes. **A** TEM images of HL-1 cardiomyocytes. The red arrows indicate damaged mitochondria. Scale bar: 10 μm. **B** Malondialdehyde (MDA) levels in cardiac tissues, *n* = 4. **C** WB showing TFR1 and MFRN1 levels in cardiac tissues, *n* = 3. **D** The concentration of Fe^2+^ in cardiac tissues, *n* = 4. **E** Fe^2+^ in HL-1 cardiomyocyte cytoplasm detected by BioTracker 575 Red Fe^2+^ Dye, *n* = 4. Scale bar: 50 μm. **F** Fe^2+^ in HL-1 cardiomyocyte mitochondria detected by Mito-FerroGreen, *n* = 4. Scale bar: 50 μm. All quantitative data are presented as mean ± SD from independent experiments. *P < 0.05, **P < 0.01, ***P < 0.001 by ordinary one-way ANOVA.

Declined GPx4 expression is a marker of ferroptosis. To detect whether ferroptosis occurred in our models, the mitochondrial morphology of HL-1 cardiomyocytes was observed through TEM. Treatment with HG and PA induced mitochondrial constriction and increased density, resembling the morphology observed after treatment with the ferroptosis inducer erastin. However, NaHS treatment significantly improved mitochondrial morphology (Fig. 3A). Furthermore, we quantified malondialdehyde (MDA) levels, which is a product of lipid ROS, to investigate the occurrence of ferroptosis in the mouse hearts. db/db mice exhibited a significant increase in MDA levels, which were inhibited by NaHS treatment (Fig. 3B). Transferrin receptor 1 (TFR1), a protein involved in iron distribution, is another specific hallmark of ferroptosis [16]. Thus, the protein levels of TFR1 and mitochondrial iron transporters mitoferrin-1 (MFRN1) were tested. We found that the levels of TFR1 and MFRN1 were both upregulated in db/db mice and restored after NaHS treatment (Fig. 3C). As a result, we observed a significant increase of free ferrous (Fe^2+^) in the cardiac tissues of db/db mice, which was restored in the NaHS-treated groups (Fig. 3D). Similarly, Fe^2+^ accumulation was observed in HL-1 cardiomyocytes treated with HG and PA. However, treatment with NaHS or Deferoxamine (DFO), an iron chelator, inhibited the accumulation of Fe^2+^ (Fig. 3E). Moreover, NaHS and DFO decreased the excessive mitochondrial Fe^2+^ levels induced by HG and PA treatment as well (Fig. 3F).

**Fig. 4:**
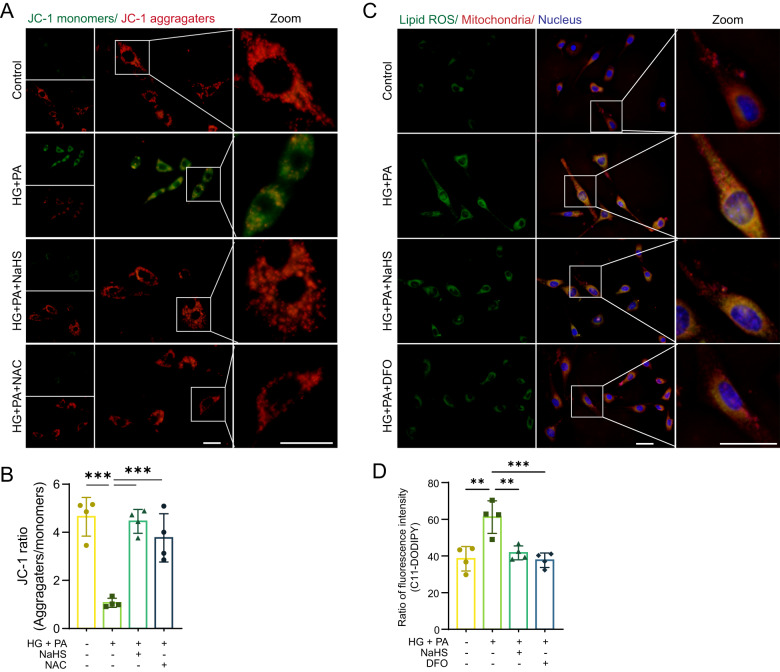
Exogenous H_2_S protects cardiac mitochondria. **A** Mitochondrial membrane potential (MMP) of HL-1 cardiomyocytes detected by fluorescence probe JC-1. Scale bar: 50 μm. **B** Quantification of JC-1, *n* = 4. **C** Lipid ROS levels and locations in HL-1 cardiomyocytes detected by C11-BODIPY. Scale bar: 50 μm. **D** Quantification of lipid ROS, *n* = 4. All quantitative data are presented as mean ± SD from independent experiments. *P < 0.05, **P < 0.01, ***P < 0.001 by ordinary one-way ANOVA.

GPx4 and GSH can also eliminate toxic lipid ROS. To examine the impact of lipid ROS on mitochondrial membranes, we utilized JC-1 and BODIPY-C11, fluorescent probes for mitochondrial membrane potential (MMP) and lipid ROS, respectively, in HL-1 cardiomyocytes. JC-1 dye enters mitochondria and undergoes reversible color change from red to green as the membrane potential decreases. Our results showed that JC-1 was present in aggregates with bright red and weak green fluorescence in the control and NaHS or NAC-treated cell groups (Fig. 4A, B). In contrast, the red fluorescent intensity in the mitochondria was significantly reduced, while the green fluorescence in the cytoplasm was significantly enhanced, indicating lower MMP in HG + PA groups (Fig. 4A, B). These findings suggest that exogenous H_2_S can maintain MMP under oxidative stress.

Interestingly, we observed the localization of lipid ROS fluorescence in mitochondria. The fluorescent intensity of lipid ROS was more pronounced in the HG + PA group compared to the control group. Treatment with NaHS or DFO significantly decreased lipid ROS levels (Fig. 4C, D).

### Exogenous H_2_S prevents apoptosis in cardiomyocytes

**Fig. 5:**
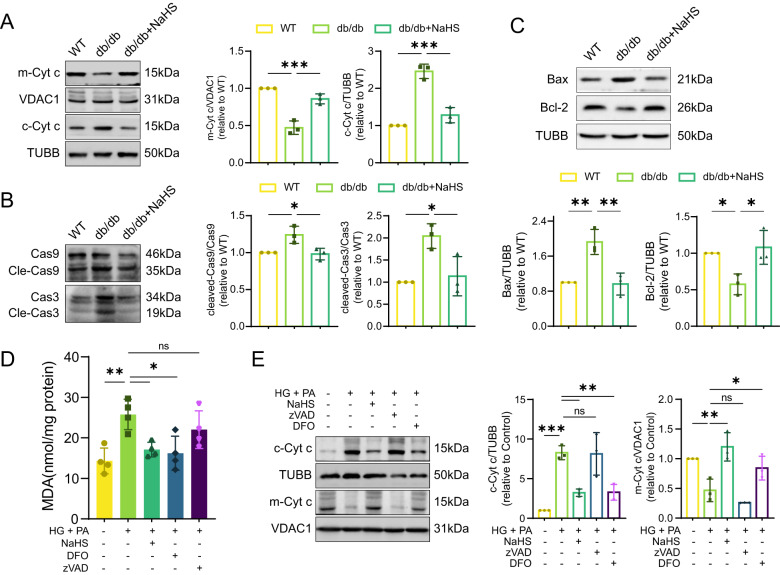
Exogenous H_2_S prevents cardiomyocytes from mitochondrial apoptosis. **A** WB showing mitochondrial cytochrome c (m-Cyt c) and cytoplasmic cytochrome c (c-Cyt c) levels in cardiac tissues, *n* = 3. **B** The protein levels of Bax and Bcl-2 in cardiac tissues, *n* = 3. **C** Cleaved-Caspase-9 (Cle-Cas9) and cleaved-Caspase-3 (Cle-Cas3) in cardiac tissues, *n* = 3. **D** MDA concentration in HL-1 cardiomyocytes, *n* = 4. **E** WB showing c-Cyt c and m-Cyt c levels of HL-1 cardiomyocytes, *n* = 3. All quantitative data are presented as mean ± SD from independent experiments. *P < 0.05, **P < 0.01, ***P < 0.001 by ordinary one-way ANOVA.

Depolarization of MMP indicated early apoptosis, accompanied by the release of mitochondrial intermembrane space protein cytochrome c, which activates initiator caspase-9 that cleaves and activates caspase-3 [17]. We hypothesized that peroxidation of mitochondrial membranes leads to cytochrome c leakage. To test this hypothesis, we next detected key proteins involved in mitochondrial apoptosis. We observed the release of mitochondrial cytochrome c, the increase of Bax, the depletion of Bcl-2, and the subsequent activation of caspase-9 and caspase-3 in the cardiac tissues of db/db mice, which were blocked by NaHS administration (Fig. 5A–C). These results indicate that exogenous H_2_S suppresses ferroptosis and mitochondrial apoptosis in db/db mouse myocardium.

To further investigate how H_2_S prevents ferroptosis and mitochondrial apoptosis in cardiomyocytes under oxidative stress, we measured MDA levels in cellular models. Stimulation with HG and PA increased MDA generation, while the addition of DFO or NaHS reduced MDA levels in cardiomyocytes. However, the addition of the apoptosis inhibitor zVAD had no effect on MDA content (Fig. 5D). Consistent with the in vivo experiments, the levels of cytochrome c in HG + PA groups were increased in the cytoplasm and decreased in the mitochondria compared to the control groups. Exogenous H_2_S or DFO inhibited the release of cytochrome c, while zVAD did not (Fig. 5E). These results suggest that ferroptosis contributes to apoptosis through promoting the leakage of mitochondrial cytochrome *c*.

### Nrf2 upregulation is a key mechanism in H_2_S-mediated cardiac protection

**Fig. 6:**
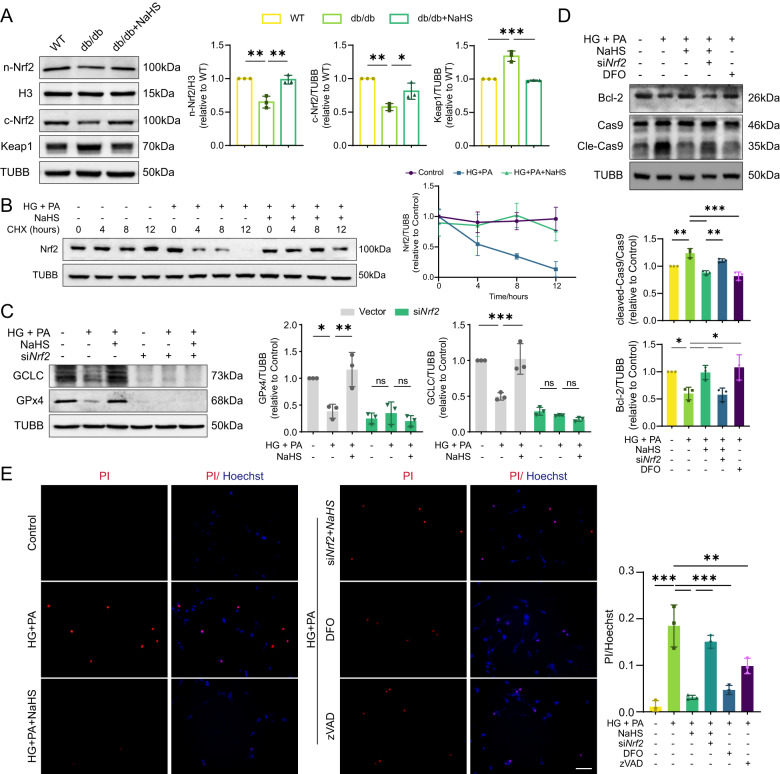
Exogenous H_2_S inhibits apoptosis through Nrf2. **A** WB showing nuclear Nrf2 (n-Nrf2), cytoplasmic Nrf2 (c-Nrf2) and Keap1 levels in cardiac tissues, *n* = 3. **B** Degradation of Nrf2 in HL-1 cardiomyocytes. Cells were treated with cycloheximide (CHX, 100 µM) at 0, 4, 8 and 12 h after other treatment, *n* = 3. **C** Protein levels of GCLC and GPx4 in HL-1 cardiomyocytes, *n* = 3. **D** The protein levels of Bcl-2 and cleavage of Cas9 in HL-1 cardiomyocytes, *n* = 3. **E** Cell death determined by PI/Hoechst staining in cardiomyocytes, *n* = 3. Scale bar: 100 μm. All quantitative data are presented as mean ± SD from independent experiments. *P < 0.05, **P < 0.01, ***P < 0.001 by ordinary one-way ANOVA.

It was found that the expression of antioxidant enzymes regulated by transcription factor Nrf2 was elevated in NaHS-treated db/db mice (Fig. 2B). To investigate the involvement of Nrf2 in cardiac damage, we examined the expression of Nrf2 and its redox sensor Keap1. Our results showed that Nrf2 levels were significantly decreased in both cytoplasm and nucleus of db/db mice, while Keap1 levels were much higher compared to WT and NaHS-treated mice (Fig. 6A). Similar results were obtained in HL-1 cardiomyocytes (Supplementary Fig. 4A). In order to explore the modulatory function of NaHS on Nrf2, cycloheximide (CHX) was given after other treatment to interfere protein synthesis at different time points. Nrf2 experienced a continuous degradation from 4 to 12 h, while NaHS treatment effectively maintained the protein’s level by enhancing its stability (Fig. 6B). Nrf2 knockdown using siRNA resulted in downregulation of GCLC and GPx4 protein levels (Supplementary Figs. 3B and 6C). Taken together, these findings suggest that NaHS treatment inhibits Nrf2 degradation, thereby improving GPx4/GSH pathway.

Furthermore, it was observed that exogenous H_2_S elevated the levels of Bcl-2 and blocked the activation of caspase-9 induced by HG and PA. However, these protective effects were abolished in cells transfected with si*Nrf2* (Fig. 6D). Treatment with DFO effectively suppressed the activation of caspase-9 (Fig. 6D). PI/Hoechst staining revealed a significant increase in the proportion of apoptotic cells in HG + PA groups and si*Nrf2*-transfected groups compared to the control and NaHS-treated groups. However, treatment with DFO or zVAD partially inhibited cell death resulted from HG + PA in HL-1 cardiomyocytes (Fig. 6E). These findings provide evidence that mitochondrial apoptosis is a downstream effect of ferroptosis, and that Nrf2 plays a crucial role in mediating the protective effects of H_2_S in diabetic cardiomyocytes.

### Exogenous H_2_S maintains Nrf2 level by regulating Syvn1-mediated Keap1 ubiquitination

**Fig. 7:**
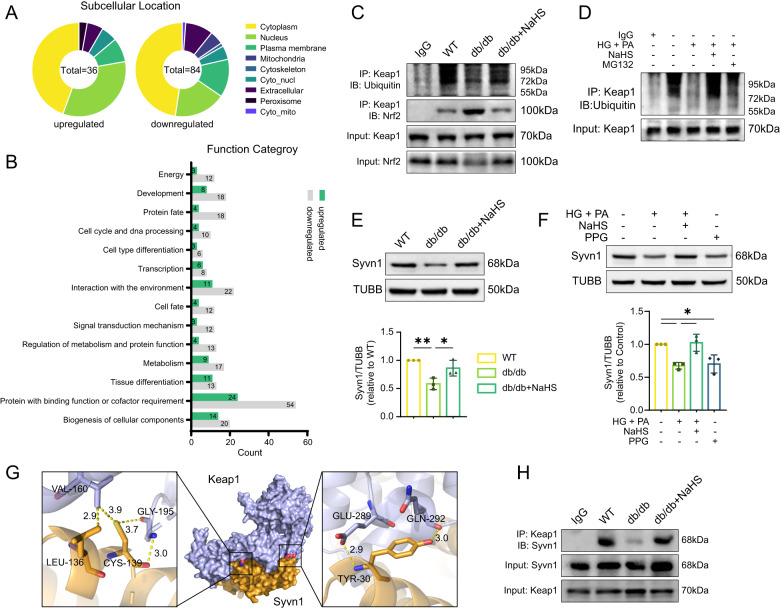
Exogenous H_2_S regulates Syvn1-mediated ubiquitination of Keap1. **A**, **B** LC-MS/MS analysis quantifying the lysine ubiquitylome of db/db and NaHS-treated db/db mice, *n* = 3. **C** Ubiquitination levels and interaction with Nrf2 of Keap1 in cardiac tissues detected by Co-immunoprecipitation (Co-IP) assay. **D** The ubiquitination levels of Keap1 in HL-1 cardiomyocytes. **E**, **F** WB showing Syvn1 levels in (**E**) cardiac tissues, *n* = 3 and in (**F**) HL-1 cardiomyocytes, *n* = 3. **G** Potential interaction sites of Keap1 and Syvn1 predicted by HDOCK and modeled by Pymol. **H** The interaction between Keap1 and Syvn1 detected by Co-IP assay. All quantitative data are presented as mean ± SD from independent experiments. *P < 0.05, **P < 0.01, ***P < 0.001 by ordinary one-way ANOVA.

mRNA sequencing analysis revealed significant alterations in gene expression associated with the ubiquitin–proteasome system in db/db and NaHS-treated mouse hearts. To further investigate the ubiquitination process, we conducted LC-MS/MS analysis to quantify the lysine ubiquitylome. The results showed that the ubiquitination levels of 36 proteins were upregulated in the NaHS-treated groups, with 16 proteins located in the cytoplasm and 12 in the nucleus (Fig. 7A). Notably, we observed an enrichment of proteins involved in binding function, cofactor requirement and environment interaction in the cardiac tissues of db/db mice compared to NaHS-treated db/db mice (Fig. 7B).

To elucidate the mechanism underlying the preservation of Nrf2 by exogenous H_2_S, we examined the ubiquitination level of Keap1. Co-immunoprecipitation (Co-IP) analysis revealed a decrease in the ubiquitination level of Keap1 in db/db mice, which resulted in Nrf2 depletion. Remarkably, exogenous H_2_S significantly increased Keap1’s ubiquitination and decreased its combination with Nrf2 (Fig. 7C). Consistently, in cellular models, the ubiquitination levels of Keap1 were also reduced under HG + PA condition, which could be reversed by NaHS (Fig. 7D). We utilized the UbiBrowser to explore potential E3 ubiquitin ligases of Keap1 [18]. Among the top-ranked candidates, we focused on synoviolin (Syvn1), a known mediator of ubiquitin transfer to substrates (Table 1). We found that Syvn1 was downregulated in the hearts of db/db mice (Fig. 7E). In cultured cells, HG + PA or PPG treatment also reduced the expression of Syvn1 (Fig. 7F). To confirm the interaction between Syvn1 and Keap1, we conducted molecular docking predictions of them and utilized HDOCK server to determine the most probable orientation (Fig. 7G). The interaction between Keap1 and Syvn1 was verified by Co-IP. The levels of Keap1 combined by Syvn1 were apparently decreased in db/db mice. Following NaHS treatment, the interaction between Keap1 and Syvn1 was enhanced due to the increased Syvn1 expression (Fig. 7H). These results suggest that depletion of Syvn1 enhances Keap1 stability, subsequently mediating Nrf2 degradation.

**Table 1:** The potential E3 ubiquitin ligases of Keap1 predicted and ranked by UbiBrowser.

ID (E3)	Gene (E3)	Go_likelihood ratio	Network_likelihood ratio	Motif_likelihood ratio	Confidence score
Q86TM6	SYVN1	1.51	1.00	6.61	0.731
O43164	PJA2	1.51	1.00	6.61	0.731
Q96AX9	MIB2	1.25	1.00	6.61	0.714
P38398	BRCA1	1.78	1.77	1.00	0.709
Q00987	MDM2	1.78	1.44	2.80	0.702
Q96PU5	NEDD4L	2.33	1.00	2.80	0.693
Q9UHC7	MKRN1	1.00	1.00	2.12	0.672

**Fig. 8:**
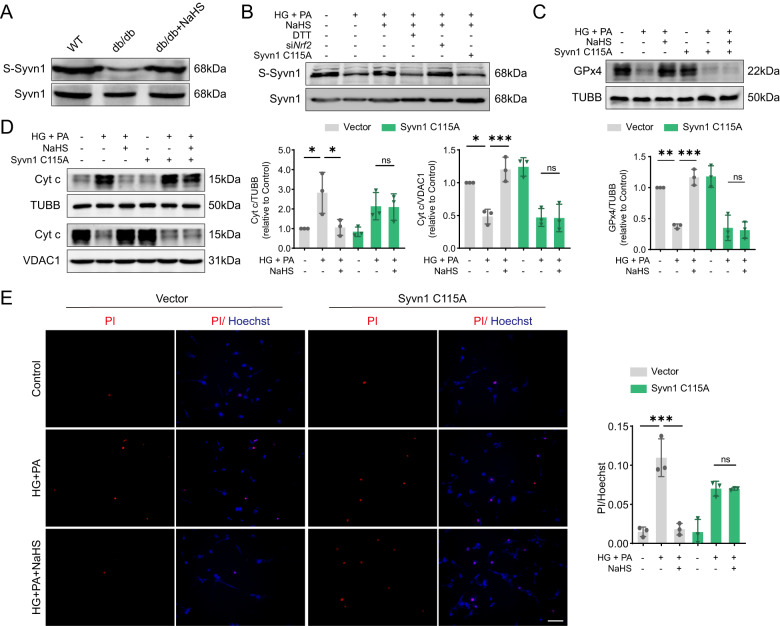
Exogenous H_2_S prevents apoptosis by regulating Syvn1-mediated ubiquitination of Keap1. **A**, **B** Sulfhydration levels of Syvn1 (S-Syvn1) in (**A**) cardiac tissues and in (**B**) HL-1 cardiomyocytes. **C** Expression of GPx4 in HL-1 cardiomyocytes, *n* = 3. **D** Cytoplasmic and mitochondrial Cyt c levels in HL-1 cardiomyocytes, *n* = 3. **E** Cell death determined by PI/Hoechst staining, *n* = 3. Scale bar: 100 μm. All quantitative data are presented as mean ± SD from independent experiments. *P < 0.05, **P < 0.01, ***P < 0.001 by ordinary one-way ANOVA.

Protein sulfhydration at cysteine sites represents a crucial regulatory mechanism of H_2_S signaling [19]. Using a modified biotin-switch assay, we identified NaHS-induced sulfhydration of Syvn1 in db/db mice (Fig. 8A). To figure out how H_2_S acted on Syvn1, we used Protein *plus* to analyze the amino acid sequence, structure and active center of Syvn1 [20]. Based on bioinformatic analysis, we modeled Syvn1 and found a key cysteine site, cys115, located in its active center (Supplementary Fig. 5A). We hypothesized that H_2_S modification of Syvn1 is dependent on cys115 site. To investigate this, we introduced a mutation where cys115 was replaced with alanine, resulting in the inhibition of H_2_S-induced sulfhydration of Syvn1 in HL-1 cardiomyocytes (Fig. 8B). Moreover, the reducing agent dithiothreitol (DTT) effectively disrupted disulfide bonds and attenuated polysulfide formation in the NaHS-treated groups. Importantly, siNrf2 had no impact on H_2_S-mediated Syvn1 sulfhydration (Fig. 8B).

Furthermore, we found that the ubiquitination level and interaction with Syvn1 of Keap1 were reduced in cells expressing the Syvn1 C115A following NaHS treatment compared to cells expressing the empty plasmid with NaHS treatment (Supplementary Fig. 5B). In the empty plasmid-transfected group, NaHS effectively mitigated the HG+PA-induced decrease in GPx4 levels. However, when we overexpressed Syvn1 C115A, this protective effect of NaHS was abolished (Fig. 8C). Besides, we investigated apoptosis markers and found that NaHS failed to preserve cytochrome c in mitochondria under HG + PA condition after transfection with the Syvn1 mutation (Fig. 8D). Consistently, exogenous H_2_S was unable to prevent apoptotic injury in cells expressing Syvn1 C115A under HG + PA condition (Fig. 8E). These results indicate that NaHS treatment promotes Syvn1-mediated ubiquitination of Keap1 to protect cardiomyocytes from mitochondrial apoptosis.

## Discussion

This study provides evidence that exogenous H_2_S administration effectively reverses cardiac damage in diabetic mice and ameliorates mitochondrial apoptosis by inhibiting ferroptosis in cardiomyocytes. Our findings demonstrate the involvement of Nrf2 in this process, as it regulates the expression of GPx4, GCLC, GCLM and GSS, both in vivo and in vitro. Additionally, we identified a novel mechanism by which exogenous H_2_S promotes Keap1 ubiquitination through modulating Syvn1, resulting in increased nuclear Nrf2. Collectively, these results unveil the Syvn1/Nrf2/GPx4 pathway as a critical mediator of the protective effects of H_2_S against ferroptosis and apoptosis induced by hyperglycemia and hyperlipidemia in cardiomyocytes.

Hyperglycemia and hyperlipidemia are the prominent metabolic abnormalities observed in type 2 diabetes mellitus, and they lead to the development of DCM [21]. In this study, db/db mice were chosen as an animal model, which characterized by hyperglycemia and hyperlipidemia. Previous studies have established a correlation between reduced left ventricular systolic and diastolic myocardial performance and excessive oxidative stress-induced cell apoptosis [22, 23]. Our echocardiography results are consistent with these findings, demonstrating alterations in cardiac function in diabetic mice.

The excessive generation of ROS causes the dysregulation of antioxidant gene expression and aberrant signal transduction, ultimately triggering cardiomyocyte death [24]. Keap1/Nrf2 plays a crucial role in reducing ROS levels by upregulating the expression of antioxidant and detoxification enzymes [25]. Exposure to ROS leads to a decline in Keap1’s ubiquitin–proteasome activity, resulting in Nrf2 stabilization and accumulation in the nucleus, where it activates its target genes. Keap1 itself undergoes various post-translational modifications, including ubiquitination, acetylation, phosphorylation and sulfhydration [26]. Recent studies have uncovered the ubiquitination of Keap1 through a proteasome-dependent pathway [27]. In our study, we observed reduced cytoplasmic and nuclear levels of Nrf2 in db/db mice and HL-1 cardiomyocytes exposed to high glucose and palmitate. Concurrently, the transcription and protein levels of GCLC, GCLM, GSS and GPx4 were also declined. Furthermore, the total GSH concentration was decreased, while the levels of oxidized GSSG and ROS were increased.

mRNA sequencing and ubiquitylome analysis between db/db and NaHS-treated db/db mice revealed the alterations in the ubiquitination degradation system in a hyperglycemic and hyperlipidemic environment. To explain the decreased Nrf2 and increased Keap1 levels in db/db mice, we identified the E3 ligase Syvn1 as a candidate through computer-based prediction and molecular docking simulations. Exogenous H_2_S was shown to regulate the expression and sulfhydration levels of Syvn1. Syvn1, a member of the tripartite motif-containing (TRIM) family with a RING motif, anchors to the ER and plays a central role in ubiquitin ligases within quality control systems, particularly in the ER-associated degradation (ERAD) system [28]. Syvn1 accepts ubiquitin from E2 ligases and transfers it to substrates, promoting their degradation [29, 30]. The active center of Syvn1 contains an essential cysteine site, cys115, which undergoes sulfhydration by H_2_S, enhancing its stability and activity. We confirmed the interaction between Syvn1 and Keap1 through Co-IP with or without the C115A mutation in Syvn1. Indeed, exogenous H_2_S enhanced Syvn1-mediated Keap1 ubiquitination through sulfhydration on the cys115 site of Syvn1, thereby maintaining Nrf2 levels and downstream gene expression.

The primary function of GPx4/GSH is to convert toxic lipid ROS products into non-toxic alcohols [31]. Deficiency of the Nrf2/GPx4 pathway has been widely implicated in the occurrence of ferroptosis [32]. Significant alterations in ferroptosis markers were observed in both in vivo and in vitro models, providing evidence for the occurrence of ferroptosis in diabetic myocardium. Furthermore, it was demonstrated that NaHS administration had the ability to ameliorate ferroptosis. In the classical ferroptosis pathway, excessive lipid ROS disrupts cell membranes, leading to regulated cell death [33]. Notably, organelle membranes, including mitochondrial membranes, are also vulnerable to ROS attack due to their similar phospholipid composition. We found partial co-localization of lipid ROS with mitochondria, suggesting potential damage to mitochondrial membranes. We detected the leakage of mitochondrial cytochrome c, the increase of Bax, the decrease of Bcl-2 and cleavage of caspase-3/9, which are markers of apoptosis. Thus, we propose that downregulation of the Nrf2/GPx4 pathway under oxidative stress induces ferroptosis and mitochondrial apoptosis.

In conclusion, our findings revealed that exogenous H_2_S promotes Syvn1-mediated Keap1 ubiquitination, leading to the modulation of the Nrf2/GPx4/GSH pathway and regulating ferroptosis accompanied with mitochondrial apoptosis in DCM. This novel mechanism sheds light on the potential therapeutic implications of H_2_S in the treatment of DCM, a multifaceted and devastating disease with limited therapeutic options. Our study provides valuable insights into the molecular mechanisms underlying the development of DCM and emphasizes the significance of exploring alternative therapeutic approaches for this disease.

## Materials and methods

### Animal experiment

Female homozygous leptin receptor-deficient (db/db) mice on a C57BL/6 background (*n* = 40) and wild-type (WT) C57BL/6 mice (*n* = 20), purchased from the Animal Laboratory Center of Nanjing University (China), were used in this study. All mice were housed in pathogen-free facilities under 22 °C, 55% humidity and 12/12 h day/night cycle. As NaHS treatment groups, 20 db/db mice were randomly selected for intraperitoneal injection of NaHS at a dose of 40 µmol/kg every 2 days from 6 weeks of age. For the animal studies, the experimenter was blinded to the group allocation during both the experiment and the outcome assessment to minimize potential biases. All animal experiments were carried out in accordance with the Guide for the Care and Use of Laboratory Animals published by the Animal Care Committees of Harbin Medical University and International Guiding Principles for Biomedical Research Involving Animals.

### Heart echocardiography

Cardiac function of mice was assessed with an echocardiography system (GE VIVID7 10 S, St. CT, Fairfield) after 20 weeks treatment of NaHS. After sedated with Avertin (240 mg/kg), mice were preconditioned by chest hair removal and placed in a supine position on an animal handling platform. Heart parameters were measured. All procedures were performed under double-blind conditions with regard to genotype or treatment.

### Cellular experiment

Cardiac Muscle Cell Line HL-1 cardiomyocytes (Sigma-Aldrich, USA) were cultured in DMEM and incubated at 37 °C in a humidified incubator with 5% CO_2_. The cell lines were authenticated recently using STR profiling before cultured and were routinely tested for mycoplasma contamination. Glucose (40 mM) and palmitate (500 µM) treatment for 48 h was used to mimic hyperglycemic and hyperlipidemic condition. We randomly divided the cultured cells into groups and the following drugs were added directly to the culture medium: NaHS (sodium hydrosulphide 100 µM, 48 h, Sigma-Aldrich), PPG (DL-propargylglycine 10 µM, 48 h, Sigma-Aldrich), NAC (N-acetyl-l-cysteine 100 µM, 48 h, Sigma-Aldrich), MG132 (20 µM, 8 h, Sigma-Aldrich), Erastin (10 µM, 12 h, MCE), DFO (Deferoxamine 10 mM, 48 h, Sigma-Aldrich), zVAD (10 µM, 12 h, MCE, USA), DTT (dithiothreitol 10 mM, 30 min, Sigma-Aldrich).

### Immunohistochemistry (IHC)

IHC was performed to evaluate the expression of CSE, CBS and 3-MST. Cardiac tissue sections were deparaffinized and rehydrated through a graded series of ethanol. Antigen retrieval was carried out using appropriate methods. Endogenous peroxidase activity was blocked by incubating the sections with 3% hydrogen peroxide. Non-specific binding was blocked using serum from the species in which the secondary antibody was raised. The sections were then incubated overnight at 4 °C with anti-CSE (Proteintech, USA, 1:200), anti-CBS (Proteintech,1:200) and anti-3-MST (Abclonal, USA, 1:200) antibodies. After washing, the sections were incubated with the corresponding secondary antibody, conjugated with a suitable enzyme such as HRP. Visualization of the protein expression was achieved using a chromogenic substrate, typically DAB. Finally, the sections were counterstained with hematoxylin, dehydrated, and mounted with a coverslip. Microscopic examination was performed, and images were captured for observation of staining intensity.

### Transmission electron microscopy (TEM)

The left ventricular heart tissues of mice were prepared for semi-thin sections and fixed in 2.5% glutaraldehyde in 0.1 M sodium phosphate buffer (pH 7.3–7.4) at 4 °C for 8 h, followed by treatment with 1% osmium tetroxide for 2 h. Tissues were then embedded in Epon 812 (Electron Microscopy Sciences, USA). The ultrathin sections were stained with uranyl acetate and lead citrate and detected under a Zeiss Axiophot microscope.

### Detection of H_2_S

The H_2_S content of HL-1 cardiomyocytes was tested with a selective H_2_S probe, 7-azido-4-methylcoumarin (C-7Az, Sigma-Aldrich). Cells were incubated with PBS containing 50 µM C-7Az at 37 °C in dark for 30 min and washed twice by PBS. The fluorescence was observed using the fluorescence microscope (Olympus XSZ-D2, Japan).

### Tissue H_2_S measurement

The left ventricular homogenates were mixed with 2 mM pyridoxal 50-phosphate, 10 mM l-cysteine and 250 mL 10% trichloroacetic acid to reach a total volume of 750 mL and incubated at 37 °C for 30 min. To trap H_2_S, 250 mL 1% zinc acetate was added. After 133 mL of 20 mM N,N dimethyl-p-phenylenedi-amine sulfate in 7.2 M HCl and 133 mL of 30 mM FeCl_3_ in 1.2 M HCl were added, the absorbance of the solution was determined at 670 nm with a spectrophotometer. The concentration of H_2_S was calculated against a calibration curve of NaHS (0.1–100 mM).

### Isolation of mitochondria in cardiac tissues and HL-1 cardiomyocytes

Mitochondria fractions of cardiac tissues and HL-1 cardiomyocytes were separated using Tissue Mitochondria Isolation kit (Beyotime, China) and Cell Mitochondria Isolation kit (Beyotime) following the manufacturer’s instructions. The final precipitate was re-suspended in mitochondrial lysis buffer or mitochondria storage buffer. The protein concentration was determined using a BCA Protein Assay kit (Solarbio, China). VDAC1 was used as a specific mitochondrial marker.

### Isolation of nucleus and membranes in cardiac tissues and HL-1 cardiomyocytes

Nucleus and membranes of cardiac tissues and HL-1 cardiomyocytes were separated using a Nucl-Cyto-Mem Preparation Kit (Applygen, China) following the manufacturer’s instructions. Nuclear and membrane fractions were then examined for protein concentration with BCA Protein Assay kit (Solarbio) and equal amounts were analyzed by western blot. Histone-H3 was used as a specific nuclear marker and CAV1 was used as a membrane marker.

### Western blot assay (WB)

WB was performed as described previously. Briefly, tissues or cells were lysed in RIPA Lysis Buffer (Beyotime) or Cell Lysis Buffer for Western and IP (Beyotime) supplemented with PMSF (Beyotime). Protein was run on a 10% or 12% SDS page gel and blotted to PVDF membrane (BioTrace, USA) by wet transfer. The blots were first incubated with anti-CSE (Proteintech, 12217-1-AP), anti-CBS (Proteintech, 14787-1-AP), anti-3-MST (Abclonal, A11587), anti-Ubiquitin (Proteintech, 10201-2-AP), anti-GPx4 (Proteintech, 67763-1-Ig), anti-GCLC (Proteintech, 12601-1-AP), anti-GCLM (Proteintech, 14241-1-AP), anti-GSS (Proteintech, 15712-1-AP), anti-TUBB (Proteintech, 10094-1-AP), anti-SLC7A11 (Proteintech, 26864-1-AP), anti-CAV1 (Proteintech, 16447-1-AP), anti-Nrf2 (Proteintech, 16396-1-AP), anti-Histone-H3 (Proteintech, 17168-1-AP), anti-caspase-3 (Proteintech, 19677-1-AP), anti-caspase-9 (Proteintech, 10380-1-AP), anti-TFR1 (Proteintech, 10084-2-AP), anti-MFRN1 (Proteintech, 26469-1-AP), anti-Cytochrome c (Proteintech, 66264-1-Ig), anti-VDAC1 (Proteintech, 55259-1-AP), anti-Keap1 (Proteintech, 10503-2-AP), anti-Syvn1 (Proteintech, 13473-1-AP), anti-Bax (Proteintech, 50599-2-Ig) and anti-Bcl-2 (Proteintech, 26593-1-AP) for 8 h at 4 °C. Secondary HPR conjugated antibody was applied for 1.5 h at room temperature. After washes in TBST for 30 min, the signal was detected by chemiluminescence. Densitometry was conducted with image processing and analysis program Image J.

### mRNA sequencing analysis

A total amount of 1 µg RNA per sample was used for the RNA sample preparations. Sequencing libraries were generated using NEBNext UltraTM RNA Library Prep Kit for Illumina (NEB) following manufacturer’s recommendations and index codes were added to attribute sequences to each sample. Briefly, mRNA was purified from total RNA using poly-T oligo-attached magnetic beads. Fragmentation was carried out using divalent cations under elevated temperature in NEBNext First Strand Synthesis Reaction Buffer (5X). First strand cDNA was synthesized using random hexamer primer and M-MuLV Reverse Transcriptase. Second strand cDNA synthesis was subsequently performed using DNA Polymerase I and RNase H. Remaining overhangs were converted into blunt ends via exonuclease/polymerase activities. After adenylation of 3’ ends of DNA fragments, NEBNext Adaptor with hairpin loop structure were ligated to prepare for hybridization. In order to select cDNA fragments of preferentially 240 bp in length, the library fragments were purified with AMPure XP system (Beckman Coulter, Beverly). Then 3 µL USER Enzyme (NEB) was used with size-selected, adaptor-ligated cDNA at 37 °C for 15 min followed by 5 min at 95 °C before PCR. Then PCR was performed with Phusion High-Fidelity DNA polymerase, Universal PCR primers and Index (X) Primer. At last, PCR products were purified (AMPure XP system) and library quality was assessed on the Agilent Bioanalyzer 2100 system. The clustering of the index-coded samples was performed on a cBot Cluster Generation System using TruSeq PE Cluster Kit v4-cBot-HS (Illumia) according to the manufacturer’s instructions. After cluster generation, the library preparations were sequenced on an Illumina platform and paired-end reads were generated.

Differential expression analysis of different groups was performed using the DESeq2. The resulting *P* values were adjusted using the Benjamini and Hochberg’s approach for controlling the false discovery rate. Genes with an adjusted *P* value < 0.05 found by DESeq2 were assigned as differentially expressed. Gene ontology (GO) enrichment analysis of the differentially expressed genes (DEGs) was implemented by the GOseq R packages based Wallenius non-central hyper-geometric distribution [34], which can adjust for gene length bias in DEGs.

### Liquid chromatography-tandem mass spectrometry (LC-MS/MS) analysis

The samples were subjected to LC-MS/MS analysis using the following procedure. Peptides were fractionated using high-pH reverse-phase high-performance liquid chromatography (HPLC) on an Agilent 300Extend C18 column (5 μm particles, 4.6 mm ID, 250 mm length). A gradient elution was performed with a mobile phase consisting of acetonitrile and 10 mM ammonium bicarbonate at pH 10. The gradient ranged from 2% to 60% acetonitrile over 80 min, resulting in the collection of 80 fractions. Subsequently, the collected fractions were consolidated into 4 fractions and dried using a vacuum centrifuge. Each fraction was then dissolved in 0.1% formic acid (FA) and loaded onto a reversed-phase pre-column (Acclaim PepMap 100, Thermo). Peptide separation was achieved using a reversed-phase analytical column (Acclaim PepMap RSLC, Thermo). The LC separation employed a gradient of solvent B (0.1% FA in 98% acetonitrile) from 6% to 22% over 22 min, followed by an increase to 36% over 8 min, and a final ramp to 80% over 5 min, which was maintained for 3 min. The flow rate was set at a constant 300 nL/min using an EASY-nLC 1000 ultra-high-performance liquid chromatography (UPLC) system. The separated peptides were subsequently analyzed using a Q ExactiveTM Plus hybrid quadrupole-Orbitrap mass spectrometer (Thermo, USA).

For mass spectrometric analysis, the peptides were subjected to nanospray ionization (NSI) and tandem mass spectrometry (MS/MS) in the Q ExactiveTM Plus instrument, which was coupled online to the UPLC system. A data-dependent acquisition method was employed, wherein one full MS scan was followed by 20 MS/MS scans. Only precursor ions with an ion count threshold of 2E4 in the MS survey scan were selected for MS/MS analysis, with 10.0 s dynamic exclusion. The electrospray voltage was set at 2.0 kV, and automatic gain control (AGC) was used to prevent ion trap overfilling, with an accumulation target of 5E4 ions for MS/MS spectra generation. The mass range for MS scans was set from 350 to 1800 *m/z*.

### Detection of ROS

The production of ROS was detected by DHE (Beyotime), DCFH (Beyotime) and MitoSOX (Invitrogen, USA). Briefly, the HL-1 cardiomyocytes were incubated with serum-free media containing DHE (25 μM), DCFH (10 μM) or MitoSOX (5 µM) for 30 min (37 °C, in dark). After incubation, cells were washed by PBS three times. Fluorescence intensity was detected using the fluorescence microscope.

### GSH/GSSG

Total glutathione and GSSG concentration in cardiac tissues and HL-1 cardiomyocytes was determined and calculated with the GSH and GSSG Assay Kit (Beyotime) following the instructions of manufacturer.

### Real-time PCR

RNA isolation and real-time PCR were performed as described previously. A qPCR SYBR Green Master Mix (Yeasen, China) was used to monitor the amplified products in real time. The changes of fluorescence of SYBR Green were tested using a LightCycler 96 System (Roche, Switzerland) and the threshold cycle (Ct) above background for each reaction was calculated. The ΔCt values were obtained from Ct values of interest genes subtracting that of loading control. The ΔΔCt values were obtained from Ct values of each sample subtracting an arbitrary calibrator. The gene expression level relative to the calibrator was expressed as 2^-ΔΔCt^. Primers:

GPX4-F, TGTGCATCCCGCGATGATT;

GPX4-R, CCCTGTACTTATCCAGGCAGA;

GCLC-F, TGCACATCTACCACGCAGTCAAG;

GCLC-R, CATCGCCTCCATTCAGTAACAAC;

GCLM-F, CCGATGAAAGAGAAGAAATGAAAGT

GCLM-R, CTCCCAGTAAGGCTGTAAATGC

GSS-F, CCCATTCACGCTTTTCCCCT;

GSS-R, GGGCAGTATAGTCGTCCTTTTTG;

TUBB-F, AGGTGCGTGAGGAGTACCC;

TUBB-R, AGGGCTTCATTGTCGATGCAG.

### siRNA transfection

HL-1 cardiomyocytes were treated according to the instructions with Nrf2 small-interfering RNA (siRNA) (Sangon Biotech, China) for 48 h to suppress Nrf2 expression. In brief, Nrf2 siRNA with the transfection reagent Lipofectamine 3000 Transfection kit (Invitrogen) were incubated for 15 min to form complexes, which were subsequently added to cells. The cells were incubated at 37 °C in a CO_2_ incubator for further treatment.

### Tissue Fe^2+^ measurement

Briefly, 10 mg cardiac tissue was homogenized in Iron Assay Buffer (Abcam, England) on ice and centrifuged at 16,000× *g* for 10 min to remove insoluble materials. The concentration of Fe^2+^ in cardiac tissues was measured using Iron Assay kit (Abcam) following the manufacturer’s instructions.

### Detection of Fe^2+^

The Fe^2+^ was detected by BioTracker 575 Red Fe^2+^ Dye (Sigma-Aldrich) and Mito- Dojindo FerroGreen (Dojindo, Japan). Briefly, the HL-1 cardiomyocytes were incubated with serum-free media containing BioTracker 575 Red Fe2+ Dye (5 µM) for 1 h or Mito-FerroGreen (5 µM) for 30 min (37 °C, in dark). After incubation, cells were washed by PBS for three times. The fluorescence intensity was detected by a fluorescence microscope.

### Assessment of mitochondrial membrane potential (MMP)

The MMP was measured by JC-1 (Beyotime). Briefly, the HL-1 cardiomyocytes were incubated with serum-free media containing JC-1 (10 µM) for 30 min (37 °C, in dark). After incubation, cells were washed by PBS for three times. The fluorescence intensity of red (aggregative JC-1) and green (monomeric JC-1) was detected by a fluorescence microscope. The ratio of red and green fluorescence intensity was calculated.

### Detection of lipid peroxidation (lipid ROS)

Lipid ROS was detected by 2 µM BODIPY 581/591 C11 (Invitrogen). After treated for 48 h, HL-1 cardiomyocytes were incubated with BODIPY 581/591 C11 for 30 min (37 °C, in dark). Cells then were washed three times by PBS and observed using the fluorescence microscope.

### Malondialdehyde (MDA) measurement

The cardiac tissues and HL-1 cardiomyocytes were homogenized and lysed in RIPA buffer on ice. Then, lysates were subjected MDA assay with MDA assay kit (Beyotimes) following the manufacturer’s instructions.

### Detection of cell death

HL-1 cardiomyocytes were stained with Hoechst (Beyotime) and PI (Solarbio) for 15 min and then detected using a fluorescence microscope. Cell death was expressed by the ratio of PI to Hoechst fluorescence intensity.

### Co-immunoprecipitation (Co-IP)

Heart tissues or HL-1 cardiomyocytes were lysed and diluted at 2 µg/µL. 320 µg protein in each sample was used for Co-IP. Protein A/G magnetic beads (Bimake, USA) were incubated with lysates mixed with antibodies (10 µg/500 µg protein) overnight at 4 °C with gentle rotation. The beads were harvested and washed three times with lysis buffer, and then were eluted into SDS Loading Buffer (Beyotime) and analyzed by WB.

### Sulfhydration assay

The assay was carried out as described previously [35]. Briefly, cardiac tissues were homogenized in 250 mM HEN buffer (250 mM Hepes, 1 mM EDTA, 0.1 mM neocuproine and 100 μM deferoxamine) and centrifuged at 13,000× *g* for 30 min at 4 °C. In total, 500 μg lysates were added to blocking buffer (HEN buffer with 2.5% SDS and 20 mM MMTS) at 50 °C for 20 min with frequent vortex. The cold acetone was added to remove the MMTS and precipitate the proteins. After precipitation at −20 °C for 1 h, the proteins were re-suspended in HEN buffer containing 1% SDS. In total, 4 mM biotin-HPDP in dimethyl sulphoxide without ascorbic acid was added to the suspension. After incubation for 3 h at 25 °C, biotinylated proteins were collected by streptavidin-agarose beads, which were then washed with HENS buffer. The biotinylated proteins were analyzed by WB.

### Point mutation of Syvn1

Adenoviruses expression GFP and Syvn1-GFP were purchased from Cyagen Biosciences Inc. The full-length mouse Syvn1 with a single mutation of C115A and GFP cDNA was inserted into pM vector between the Kozak and T2A sites. After transfection with adenovirus to HL-1 cardiomyocytes for 8–12 h, new fresh medium was added. Cells were treated with different reagents 12 h later, and the related proteins were examined by western blot.

### Statistics

The determination of sample size aims to ensure adequate statistical power for detecting a predetermined effect size. This involves estimating the expected effect size through prior research, literature review, or expert opinions.

We excluded mice in which diabetes modeling was unsuccessful through blood glucose and glucose tolerance testing. Furthermore, extreme outlier samples were excluded from the analysis to minimize potential biases and confounding factors, ensuring the study remains focused on the intended research objectives.

The data were examined to assess whether they met the assumptions of the selected statistical tests. The data were analyzed by Prism software package (GraphPad Software). Results were expressed as the mean ± standard deviation (SD). Two groups were compared using unpaired *t* test, while more than two groups were compared by ordinary one-way ANOVA and Tukey correction. The investigator was blinded to the group allocation during outcome assessment to minimize potential bias.

### Supplementary information

Supplementary legend

Supplementary Figure 1

Supplementary Figure 2

Supplementary Figure 3

Supplementary Figure 4

Supplementary Figure 5

Original western blots

## Data Availability

The data presented in this study are available on request from the corresponding authors.

## Study approval

The manuscript does not contain clinical studies or patient data. The animal study protocol was approved by the Institutional Research Board of Harbin Medical University (protocol code HMUIRB2022015, 30 September 2022).
