# Supplementary material for: Exogenous H2S initiating Nrf2/GPx4/GSH pathway through promoting Syvn1-Keap1 interaction in diabetic hearts
Source: Cell Death Discov. 2023 Oct 24;9:394. doi: 10.1038/s41420-023-01690-w (PMC10598017; doi:10.1038/s41420-023-01690-w)
Supplement: Supplementary file 1 — Supplementary legend [file 41420_2023_1690_MOESM1_ESM.docx]

**Supplementary Figure 1.** Basic parameters of animal models. **(A)** Body weight and blood glucose, n=6. **(B)** Glucose tolerance, n=6. All quantitative data are presented as mean ± SD from independent experiments. *p < 0.05, **p < 0.01, ***p < 0.001 by ordinary one-way ANOVA.

**Supplementary Figure 2.** Exogenous H_2_S improves the antioxidant system of cardiomyocytes. **(A)** mRNA levels of *Gpx4*, *Gclc*, *Gclm* and *Gss* in cardiac tissues, n=3 or 4. **(B)** Expression of GPx4, GCLC, GCLM, GSS and SLC7A11 in HL-1 cardiomyocytes, n=3. All quantitative data are presented as mean ± SD from independent experiments. *p < 0.05, **p < 0.01, ***p < 0.001 by ordinary one-way ANOVA.

**Supplementary Figure 3.** ROS levels of cardiomyocytes. **(A-C)** ROS levels in HL-1 cardiomyocytes detected by fluorescence probes **(A)** DCFH, n=4, **(B)** DHE, n=3 and **(C)** MitoSOX, n=3. Scale bar: 50 μm. All quantitative data are presented as mean ± SD from independent experiments. *p < 0.05, **p < 0.01, ***p < 0.001 by ordinary one-way ANOVA.

**Supplementary Figure 4.** Exogenous H_2_S preserves Nrf2 in cardiomyocytes. **(A)** Nuclear Nrf2, cytoplasmic Nrf2 and Keap1 levels in HL-1 cardiomyocytes, n=3. **(B)** Protein levels of Nrf2 in HL-1 cardiomyocytes, n=3. All quantitative data are presented as mean ± SD from independent experiments. *p < 0.05, **p < 0.01, ***p < 0.001 by ordinary one-way ANOVA.

**Supplementary Figure 5.** Exogenous H_2_S modulates ubiquitination and Sulfhydration. **(A)** Prediction of the active center of Syvn1 using Protein *plus* and Pymol*.* **(B)** The ubiquitination levels and interaction with Syvn1 of Keap1 in HL-1 cardiomyocytes.
