# Supplementary material for: Exogenous H2S initiating Nrf2/GPx4/GSH pathway through promoting Syvn1-Keap1 interaction in diabetic hearts
Source: Cell Death Discov. 2023 Oct 24;9:394. doi: 10.1038/s41420-023-01690-w (PMC10598017; doi:10.1038/s41420-023-01690-w)
Supplement: Supplementary file 7 — Original western blots [file 41420_2023_1690_MOESM7_ESM.pdf]

Manuscript number: CDDISCOVERY-23-1322

This file contains all the bands that appear in our Figures. To enhance the visual clarity and comprehensibility of the Figures, we have chosen to present selected bands (indicated by boxed annotations) in the respective figures. However, during the quantitative analysis (depicted in the statistical graphs), each band is normalized to its own internal control and compared to the corresponding control group for accurate representation.

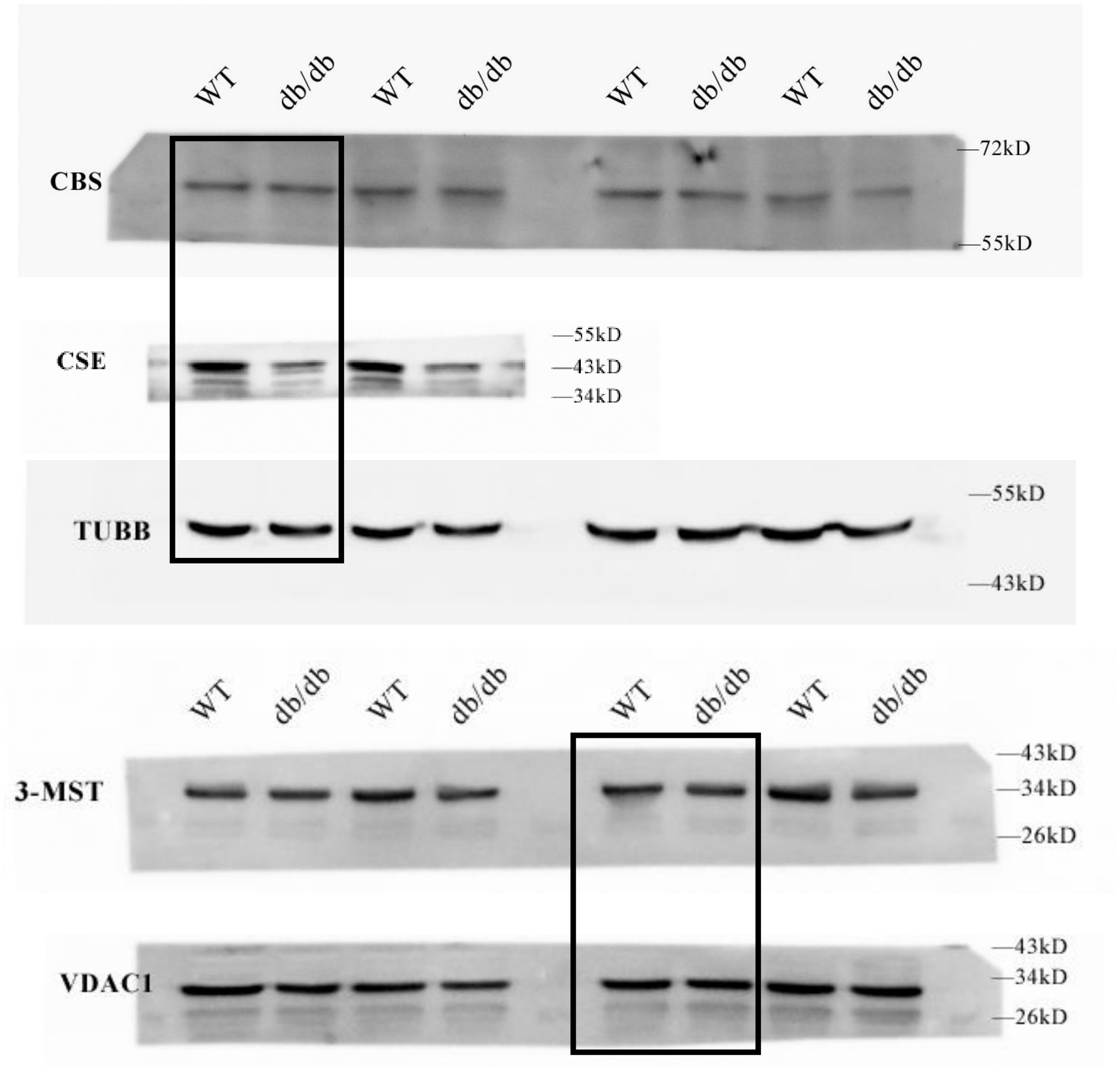

Full unedited gel for Figure 1B

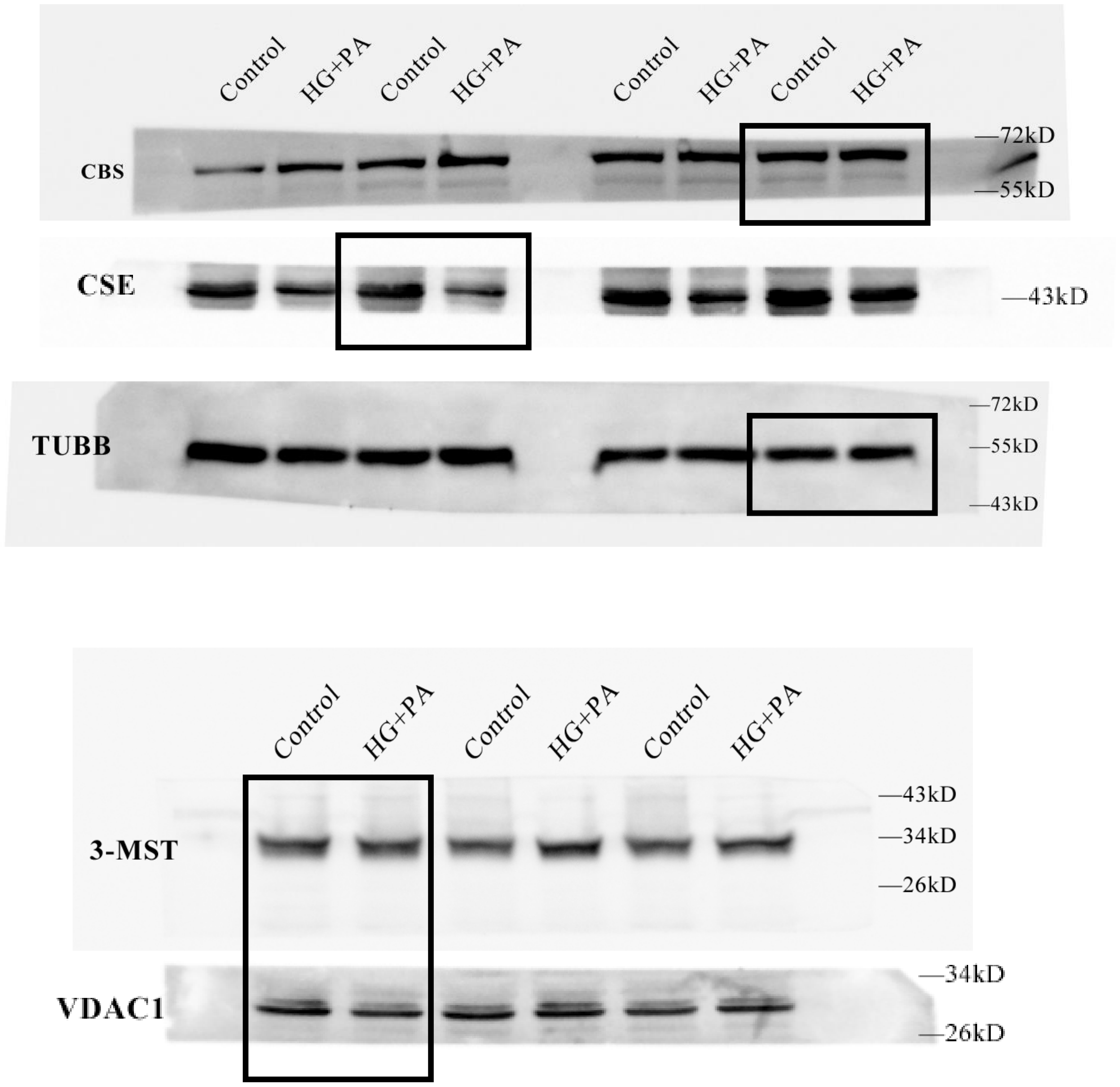

Full unedited gel for Figure 1D

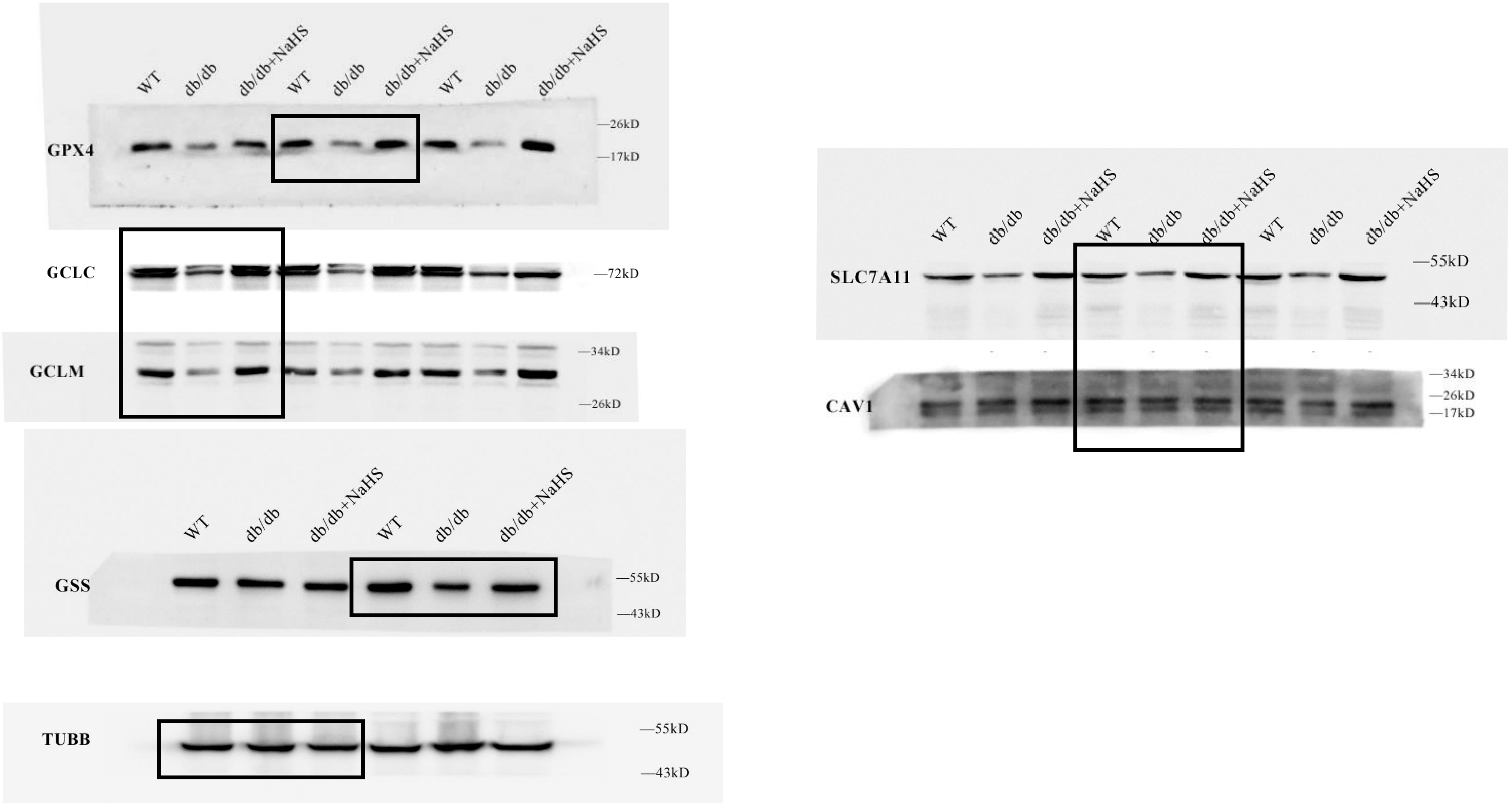

Full unedited gel for Figure 2E

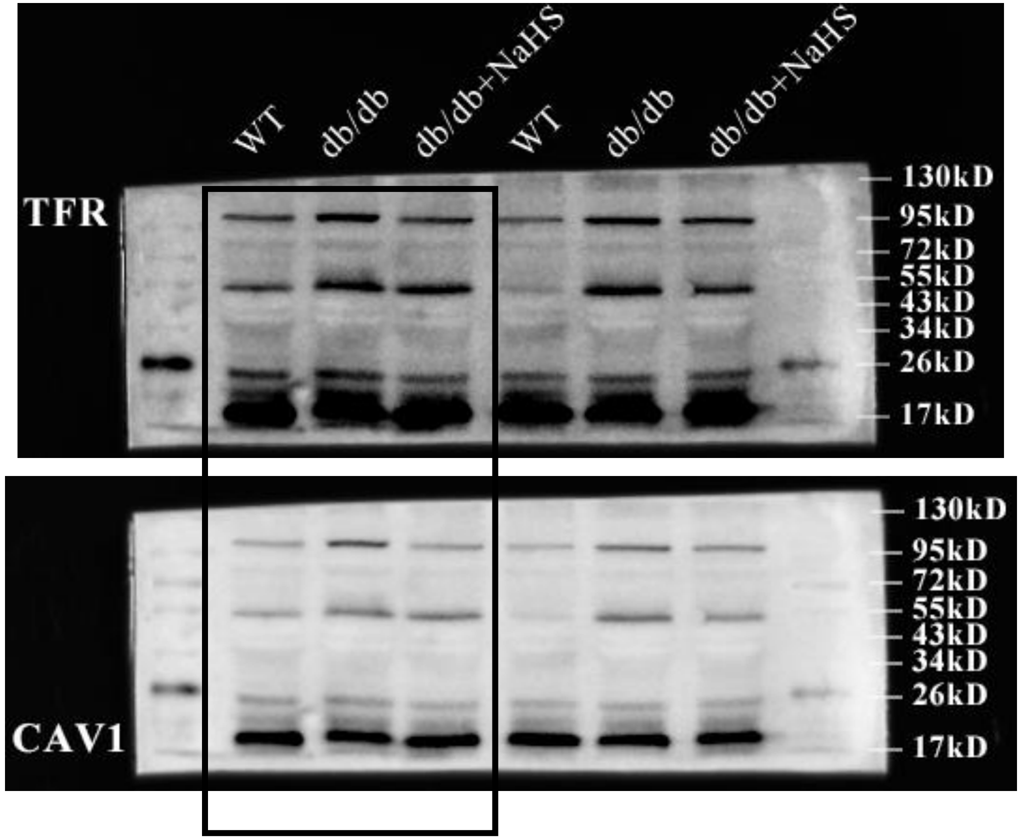

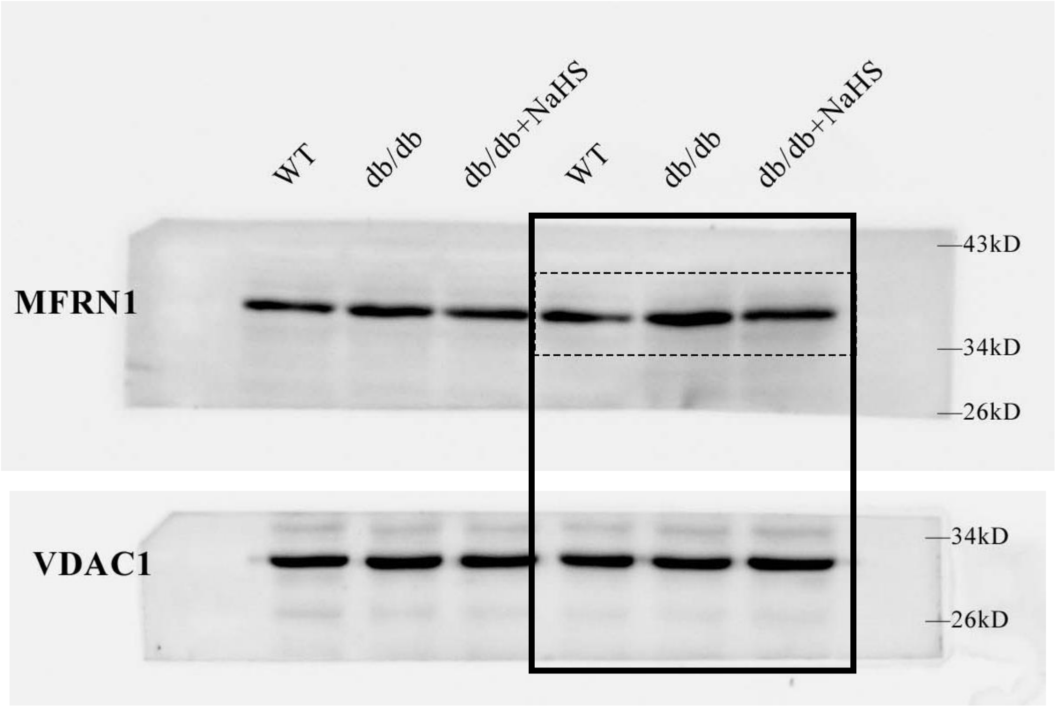

Full unedited gel for Figure 3C

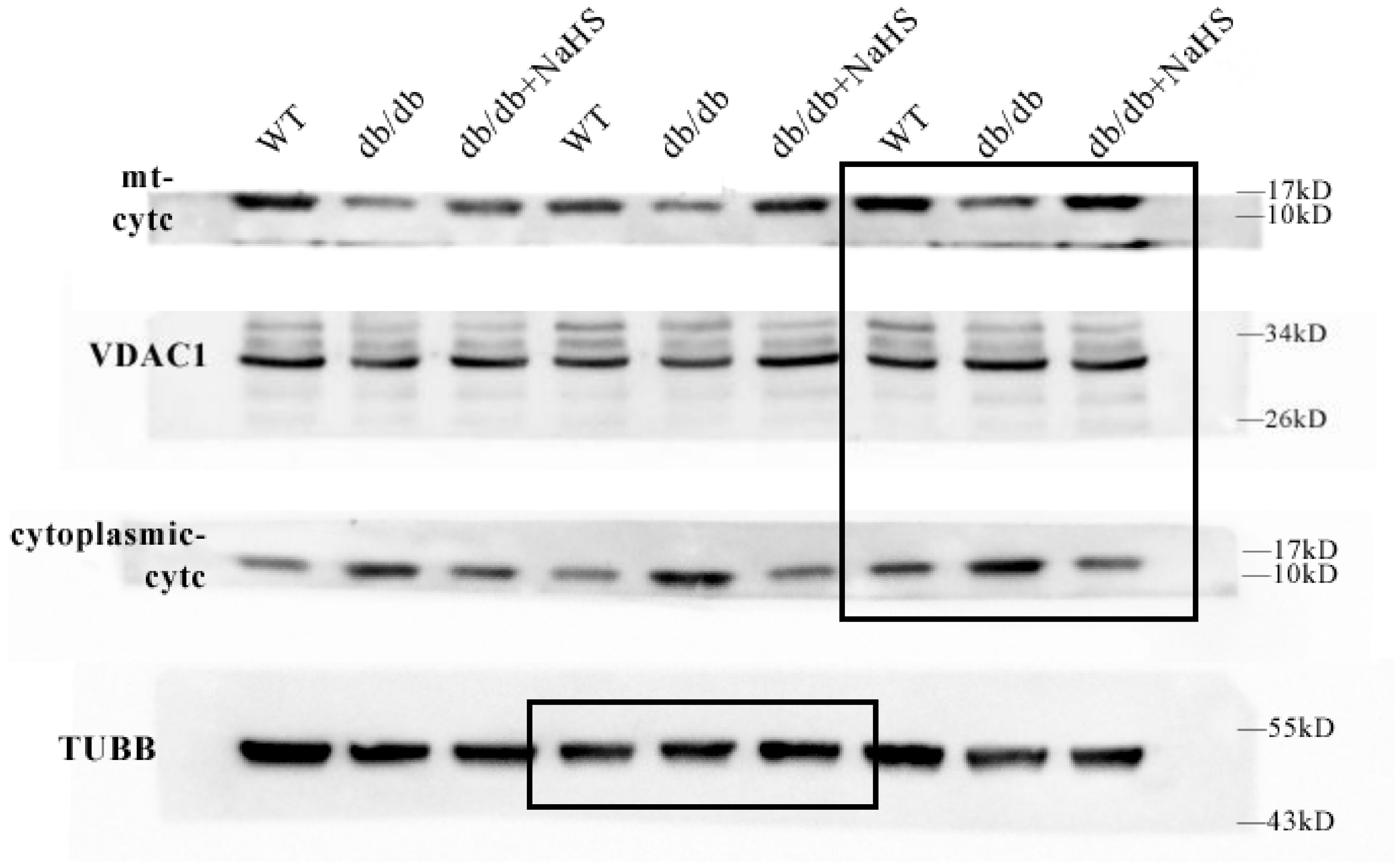

Full unedited gel for Figure 5A

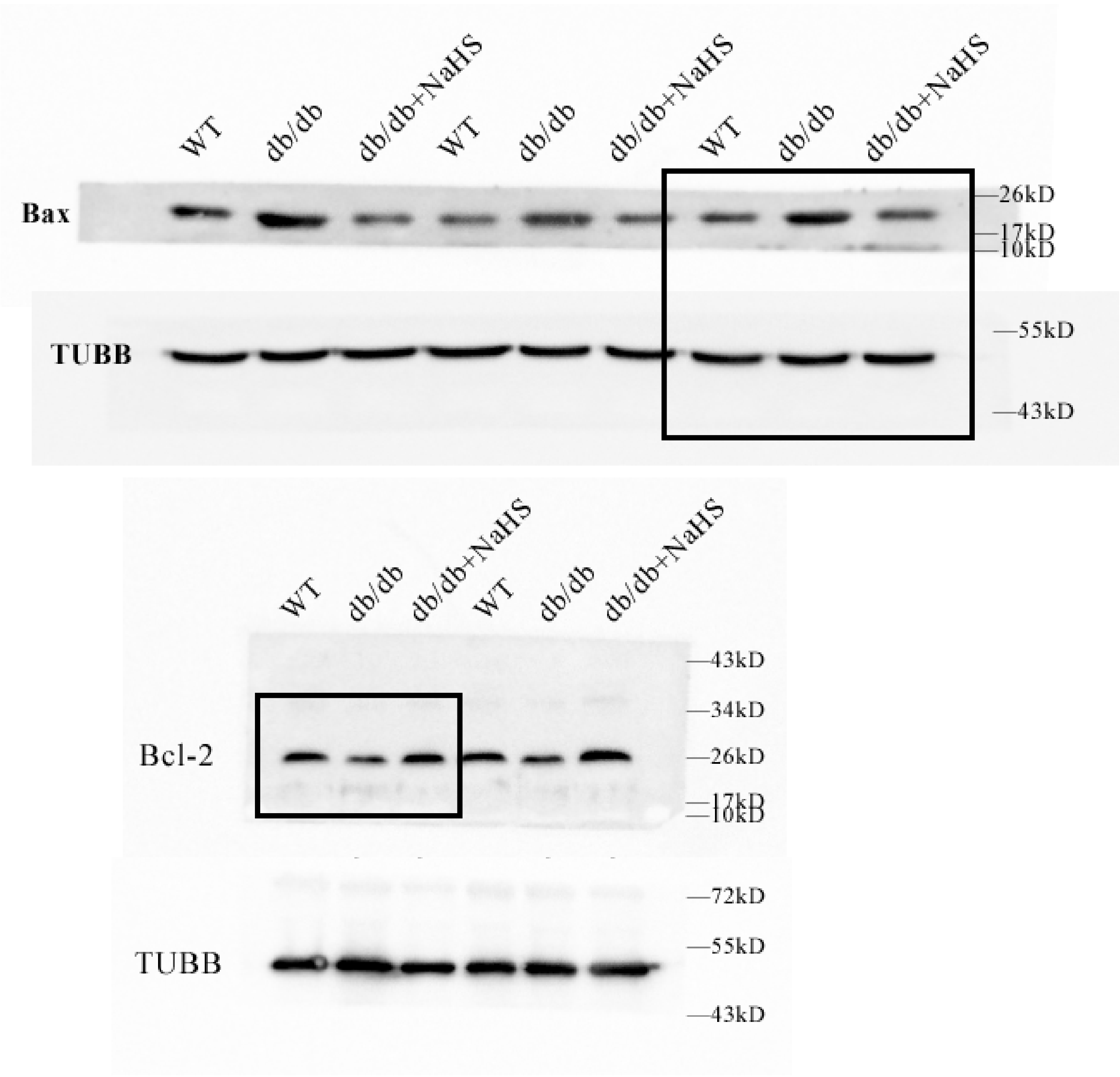

Full unedited gel for Figure 5B

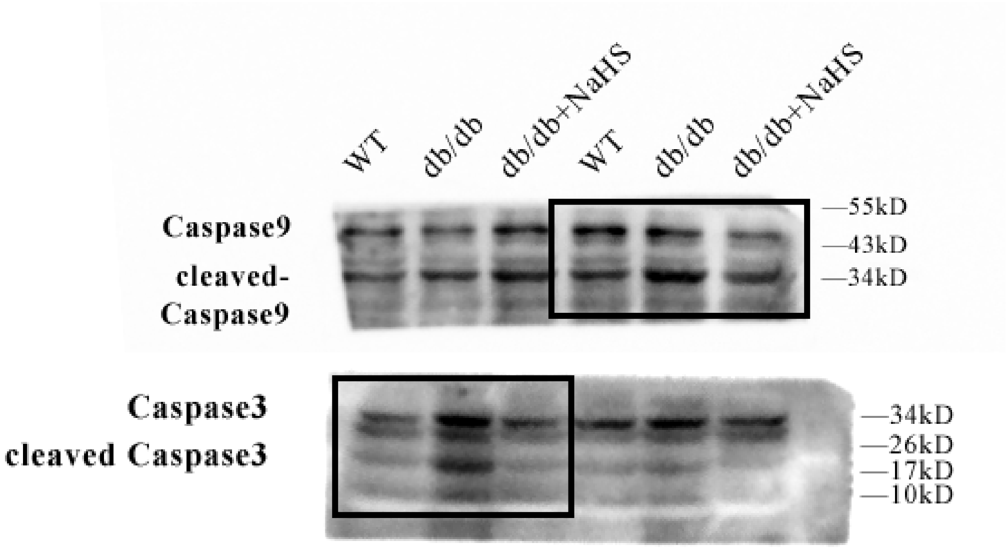

Full unedited gel for Figure 5C

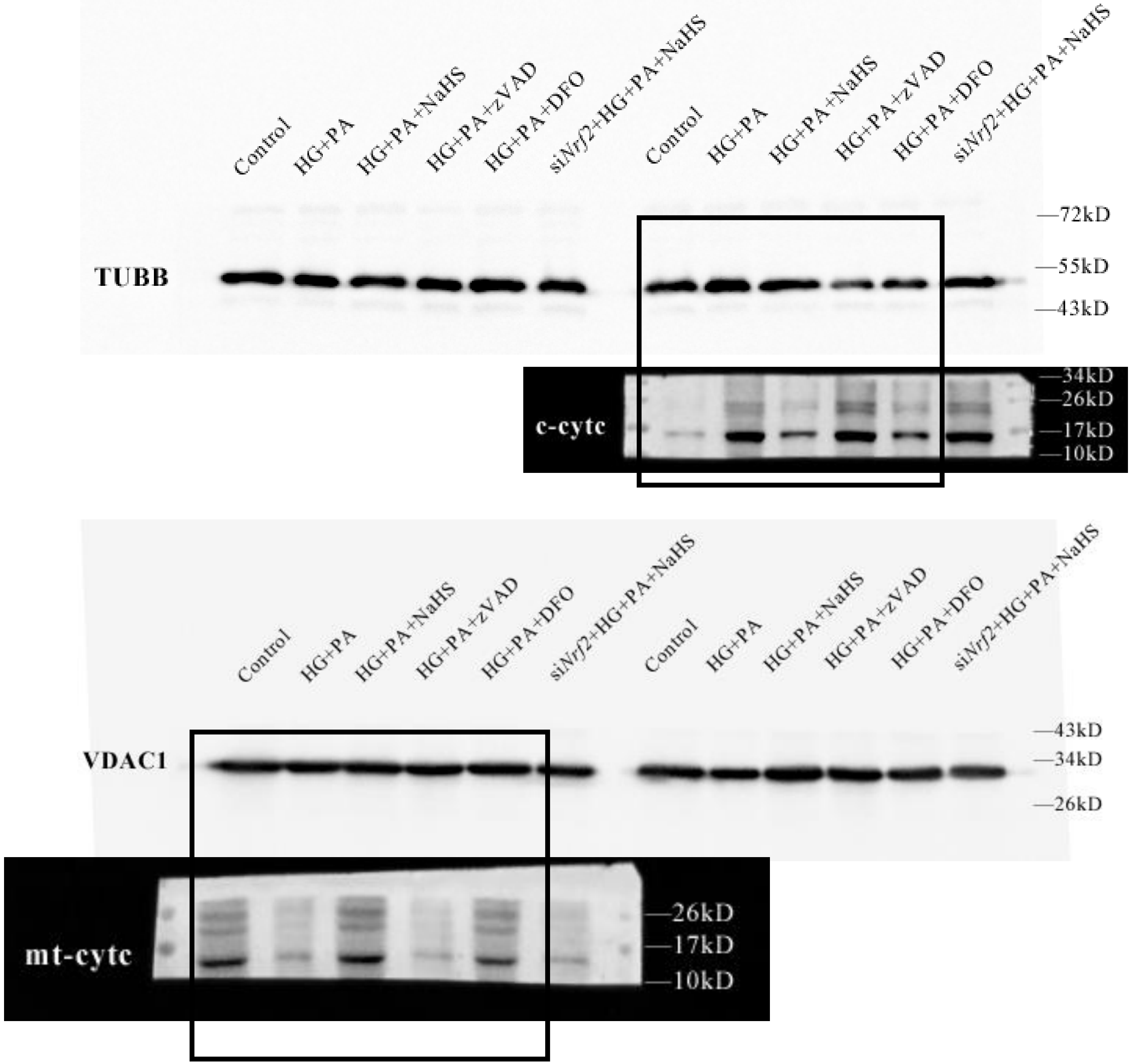

Full unedited gel for Figure 5E

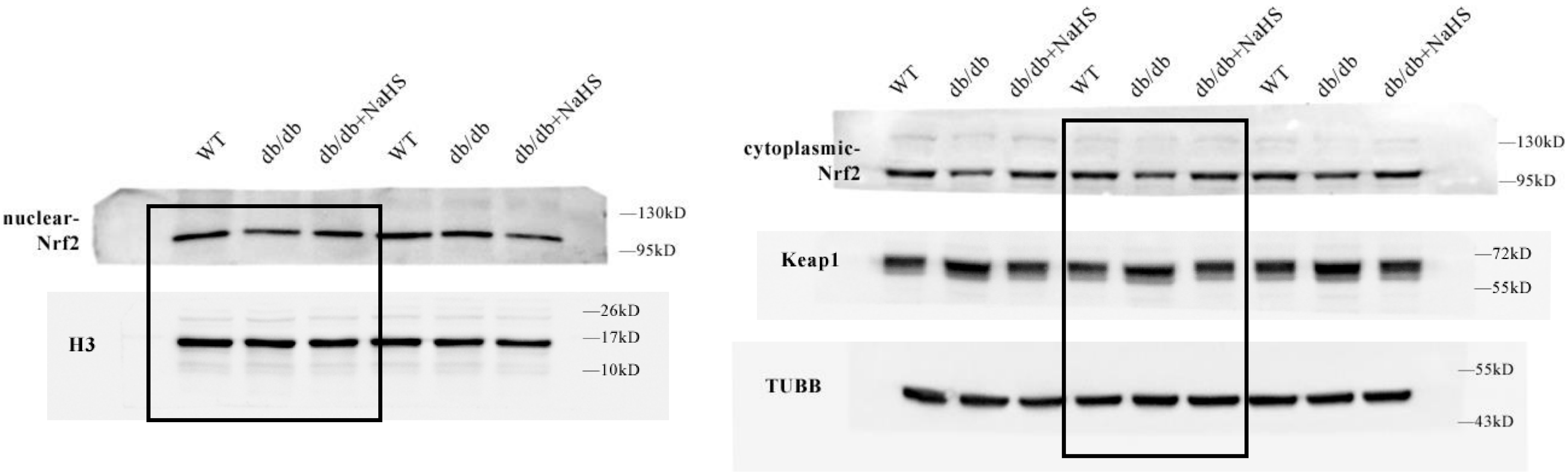

Full unedited gel for Figure 6A

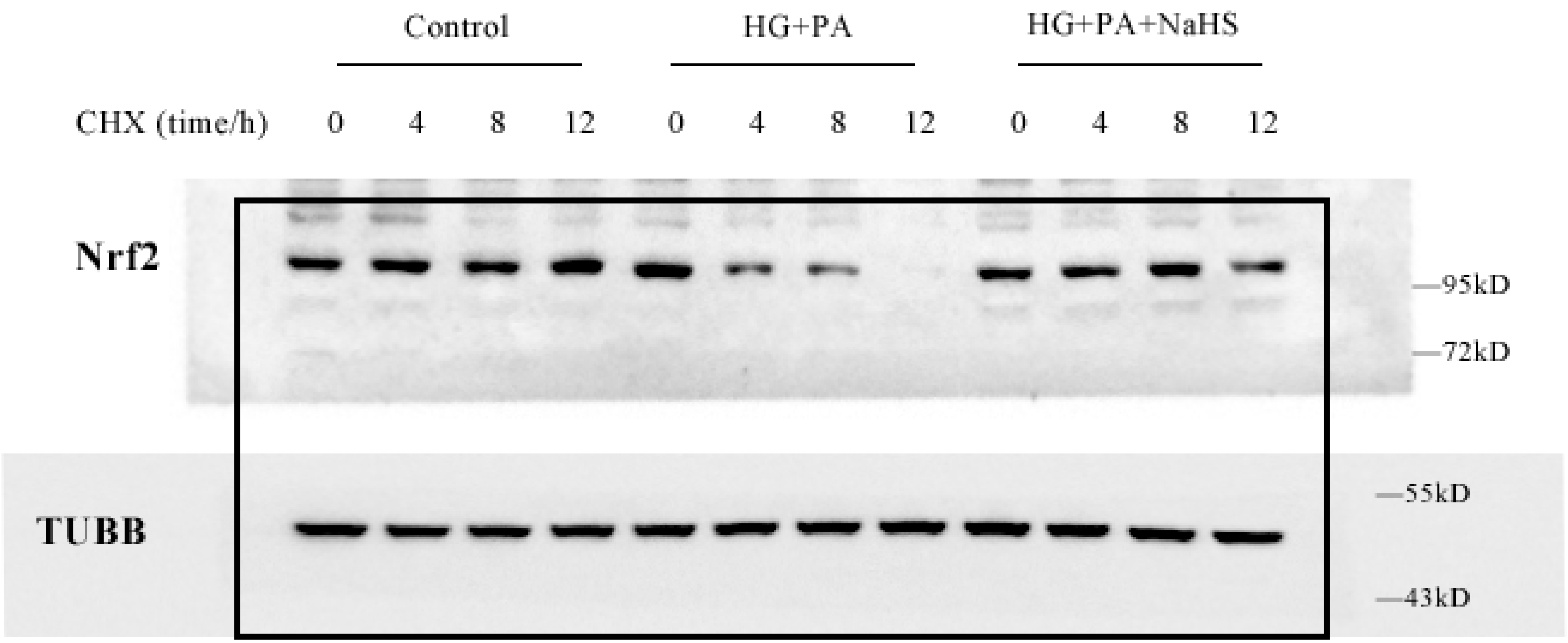

Full unedited gel for Figure 6B

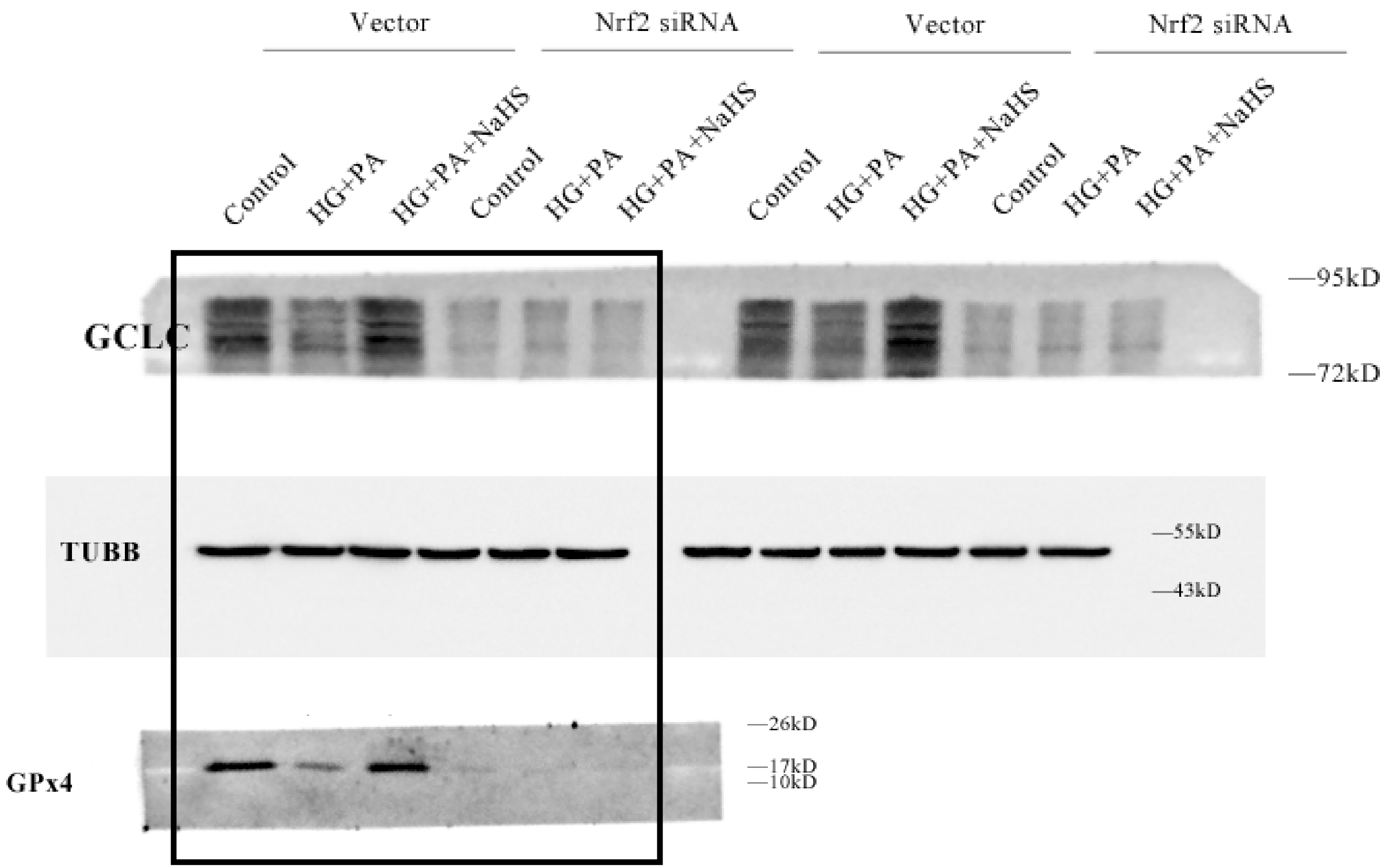

Full unedited gel for Figure 6C

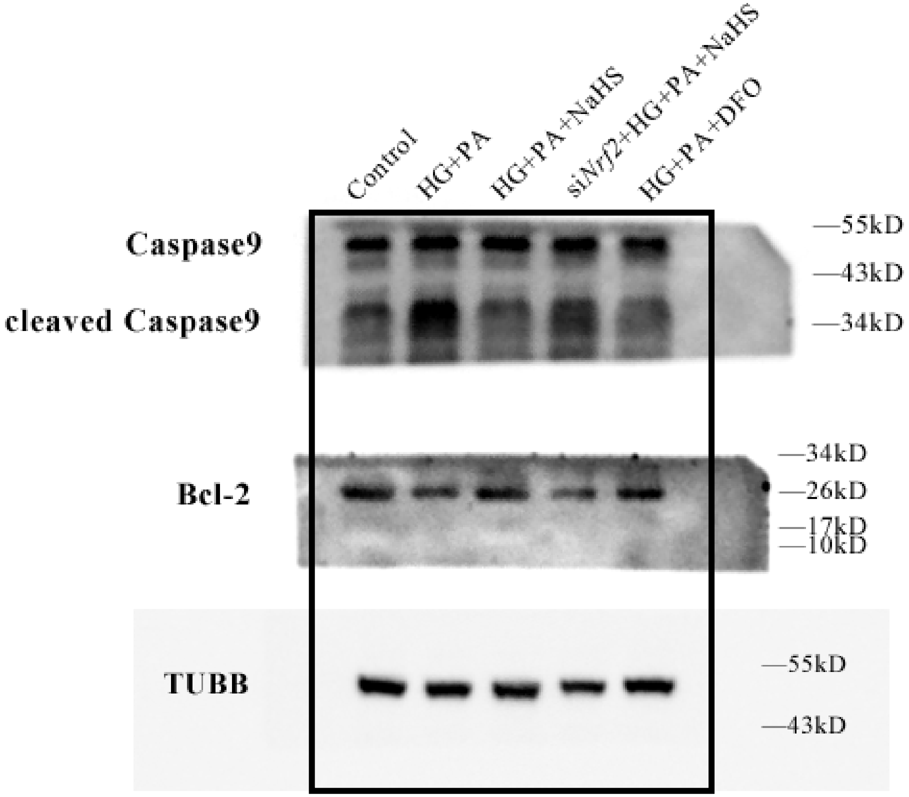

Full unedited gel for Figure 6D

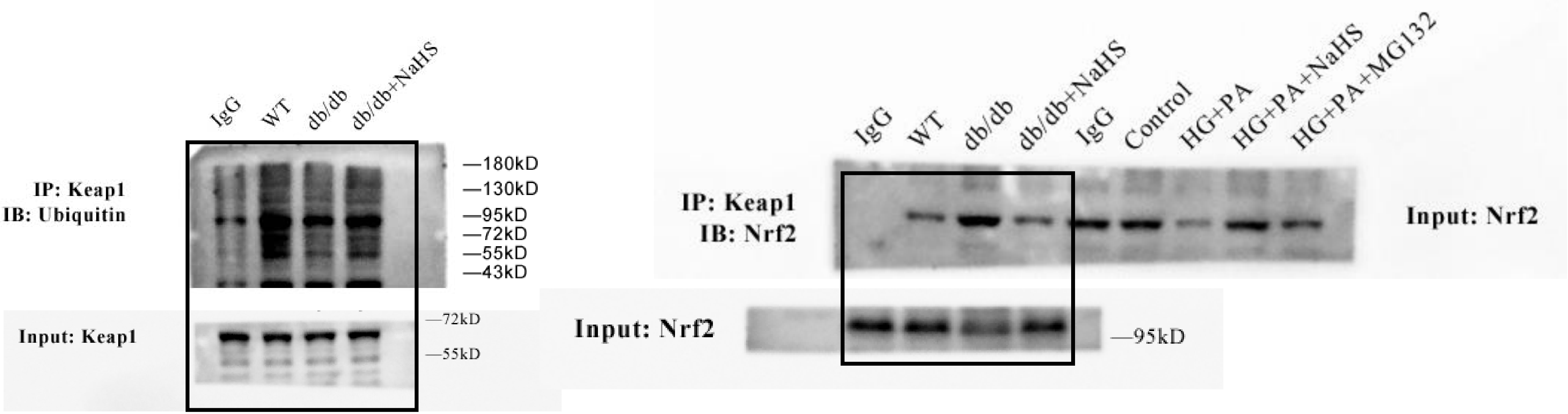

Full unedited gel for Figure 7C

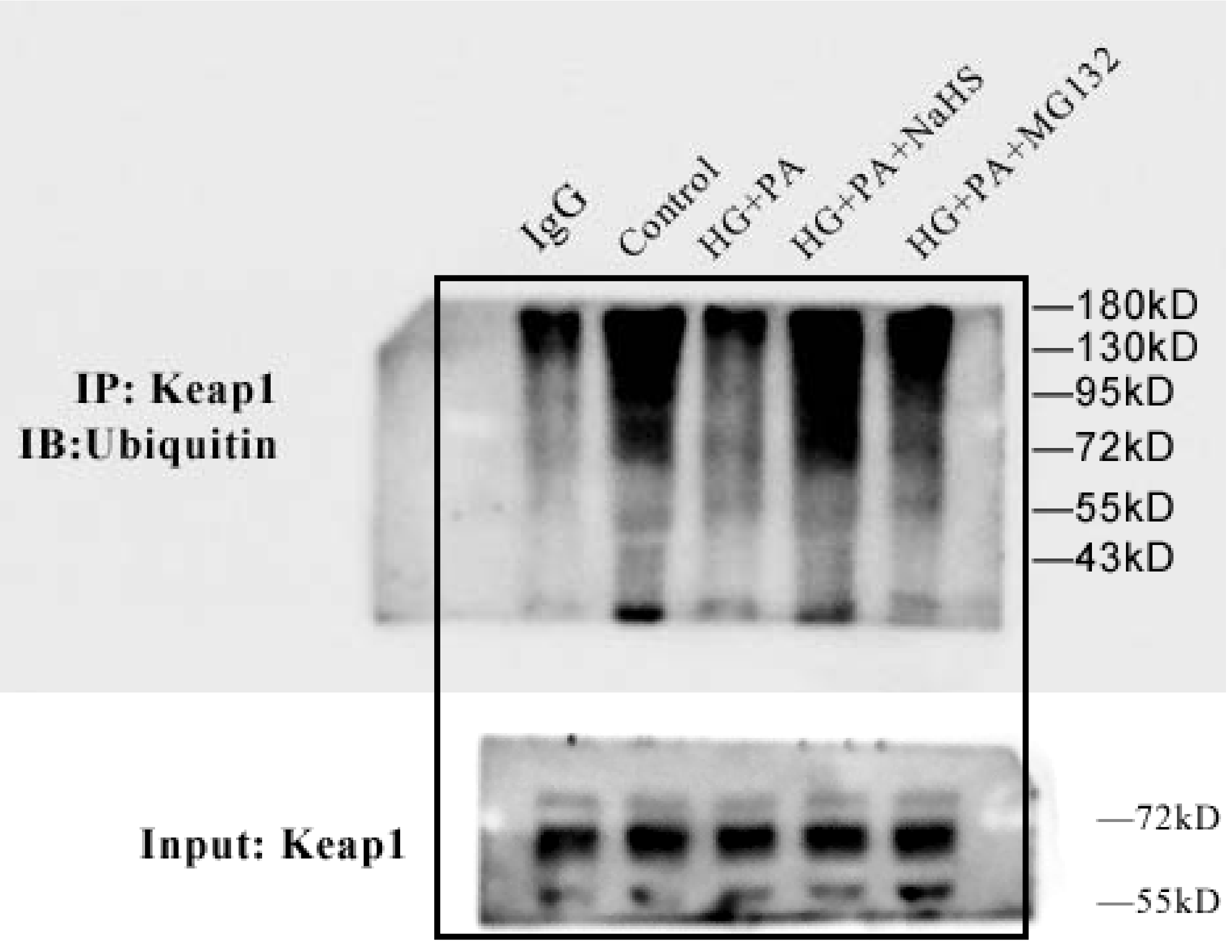

Full unedited gel for Figure 7D

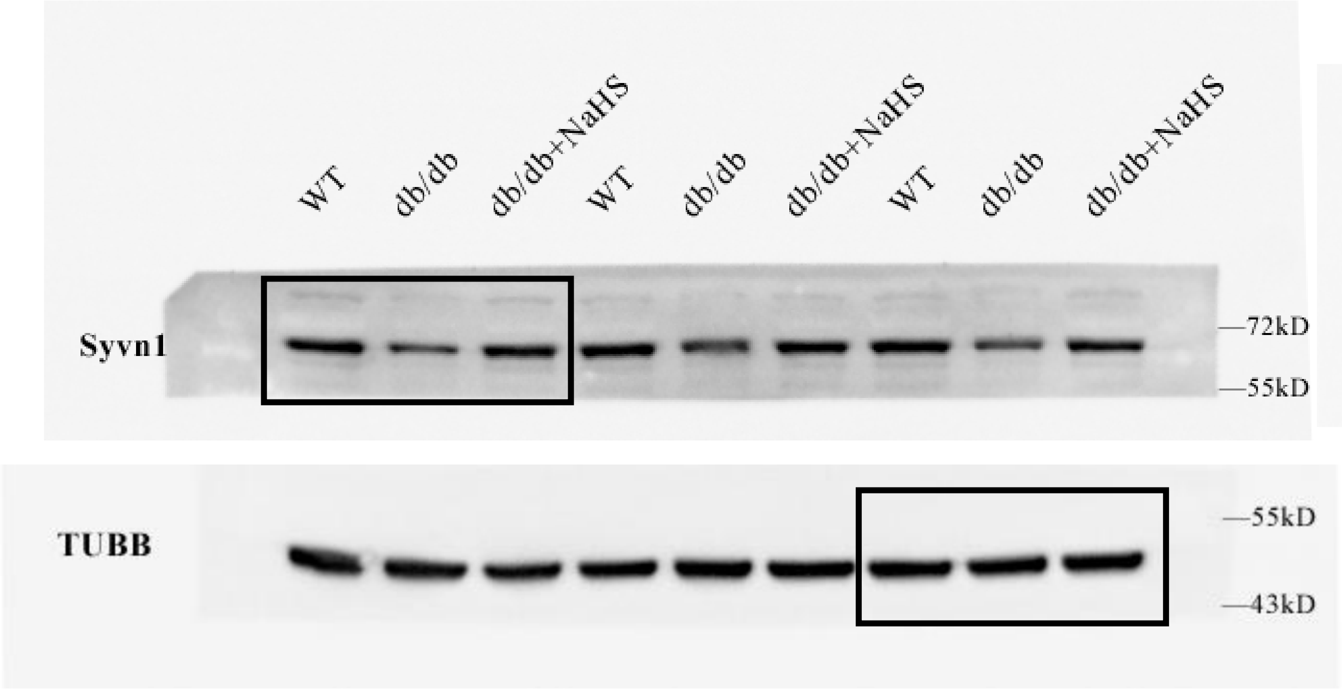

Full unedited gel for Figure 7E

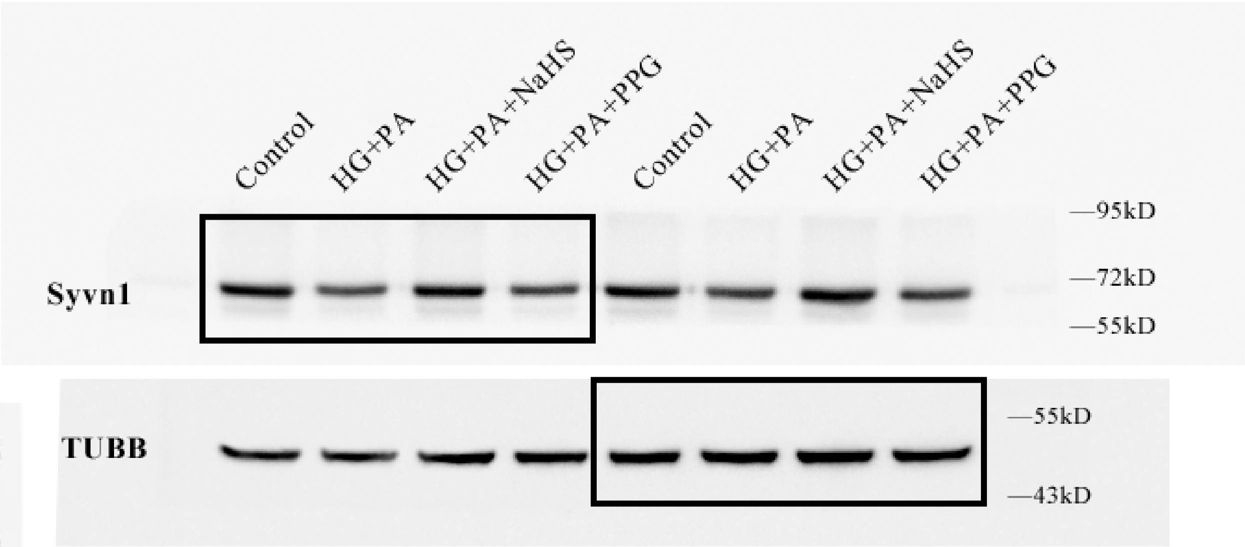

Full unedited gel for Figure 7F

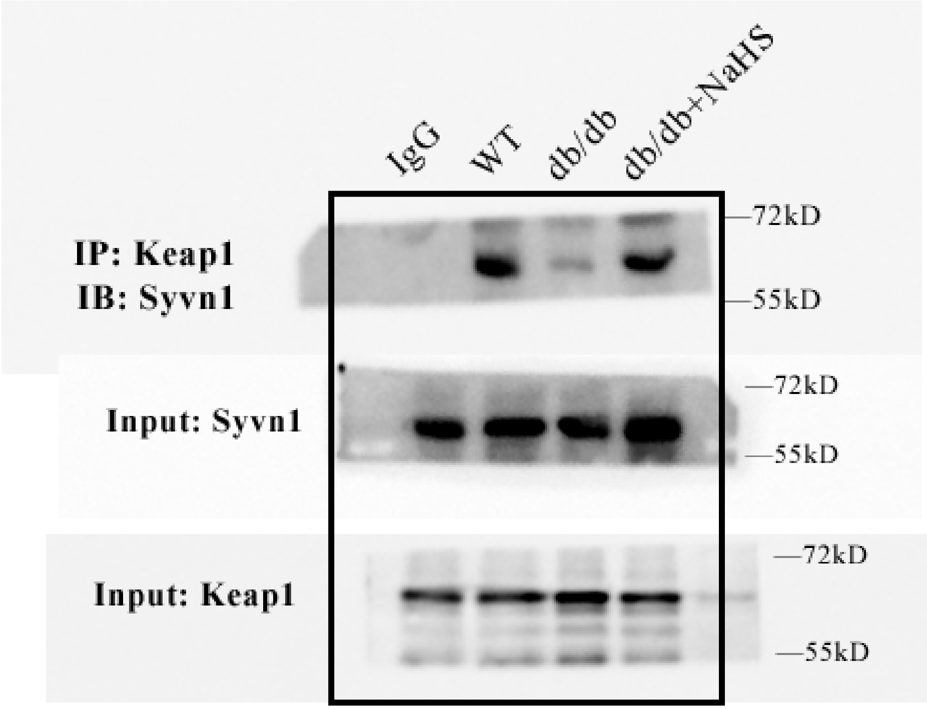

Full unedited gel for Figure 7H

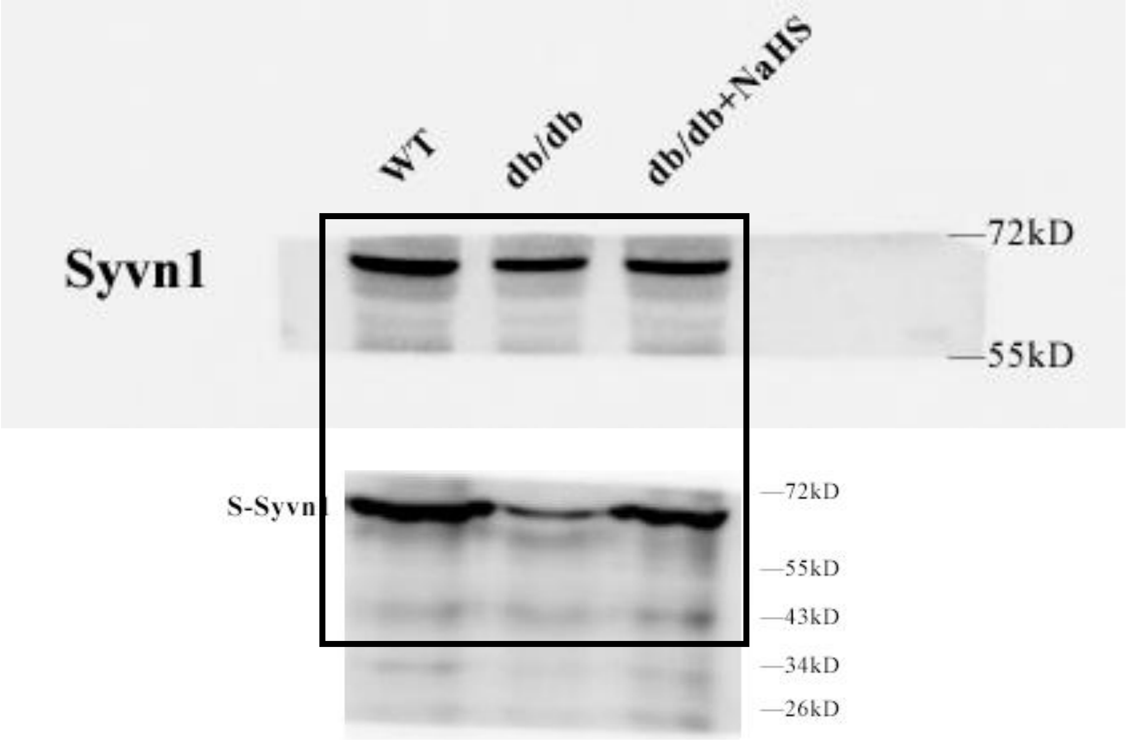

Full unedited gel for Figure 8A

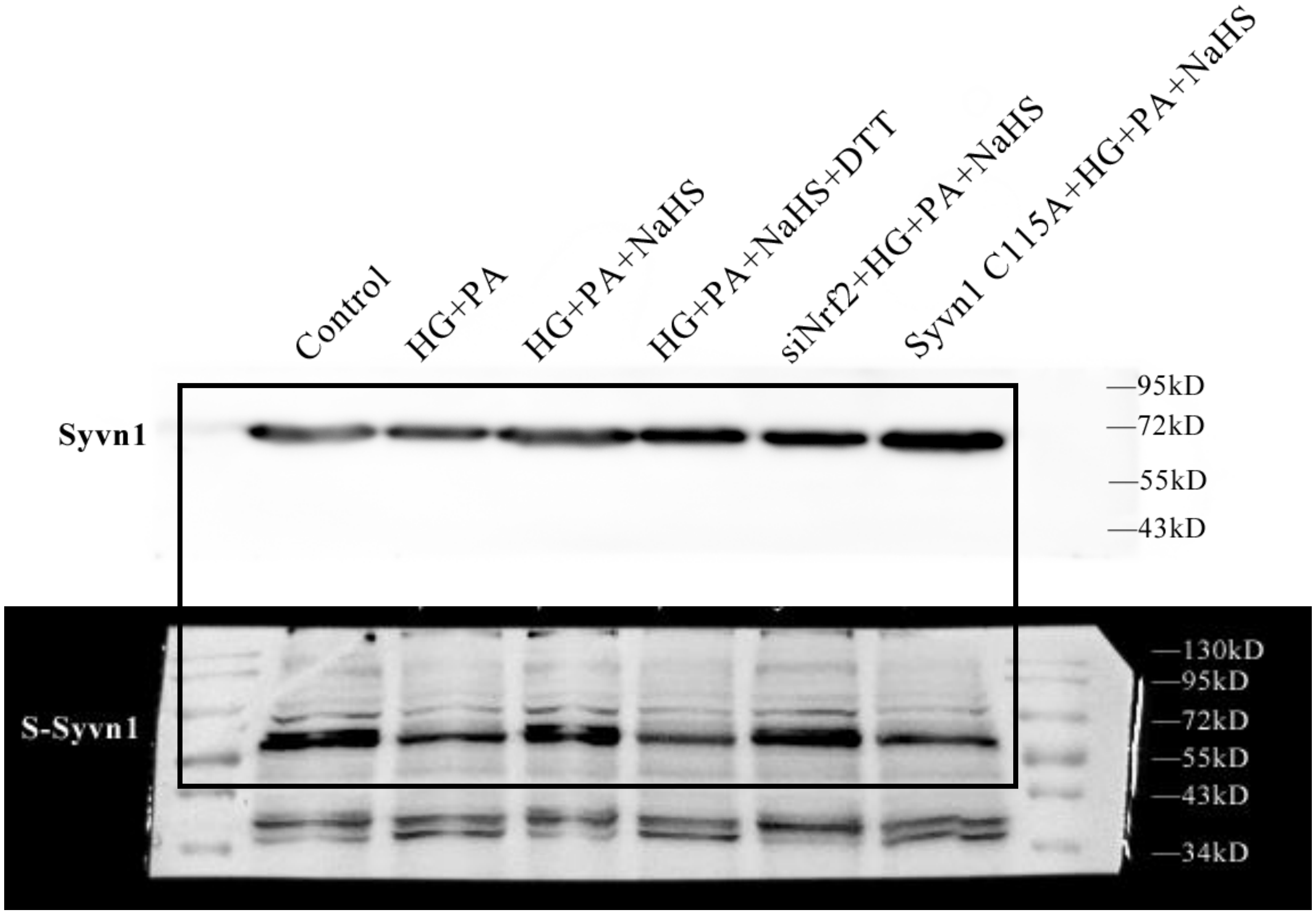

Full unedited gel for Figure 8B

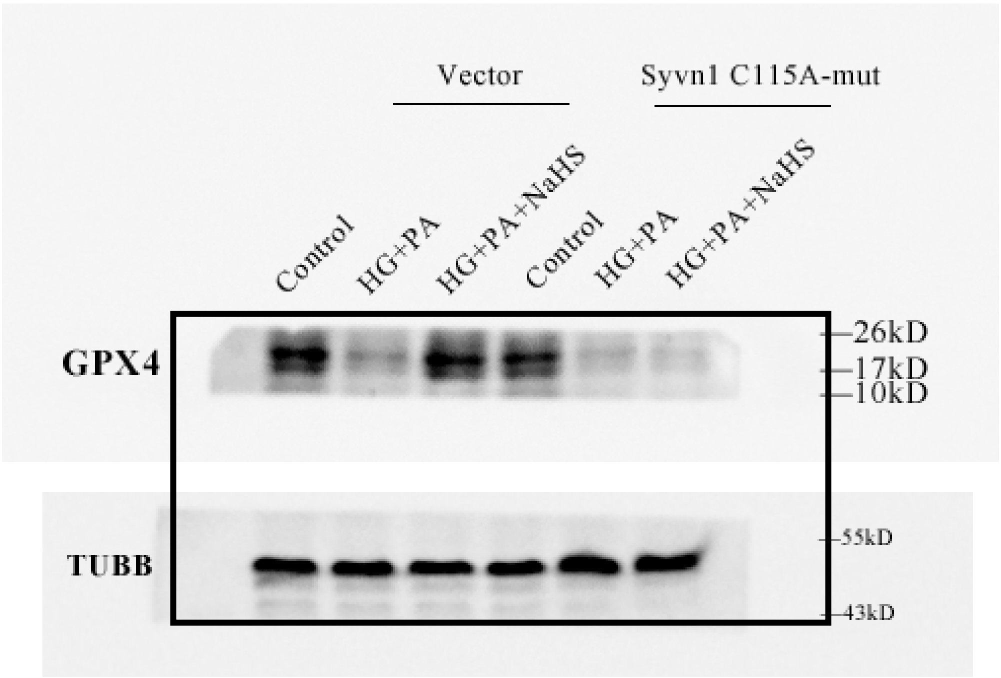

Full unedited gel for Figure 8C

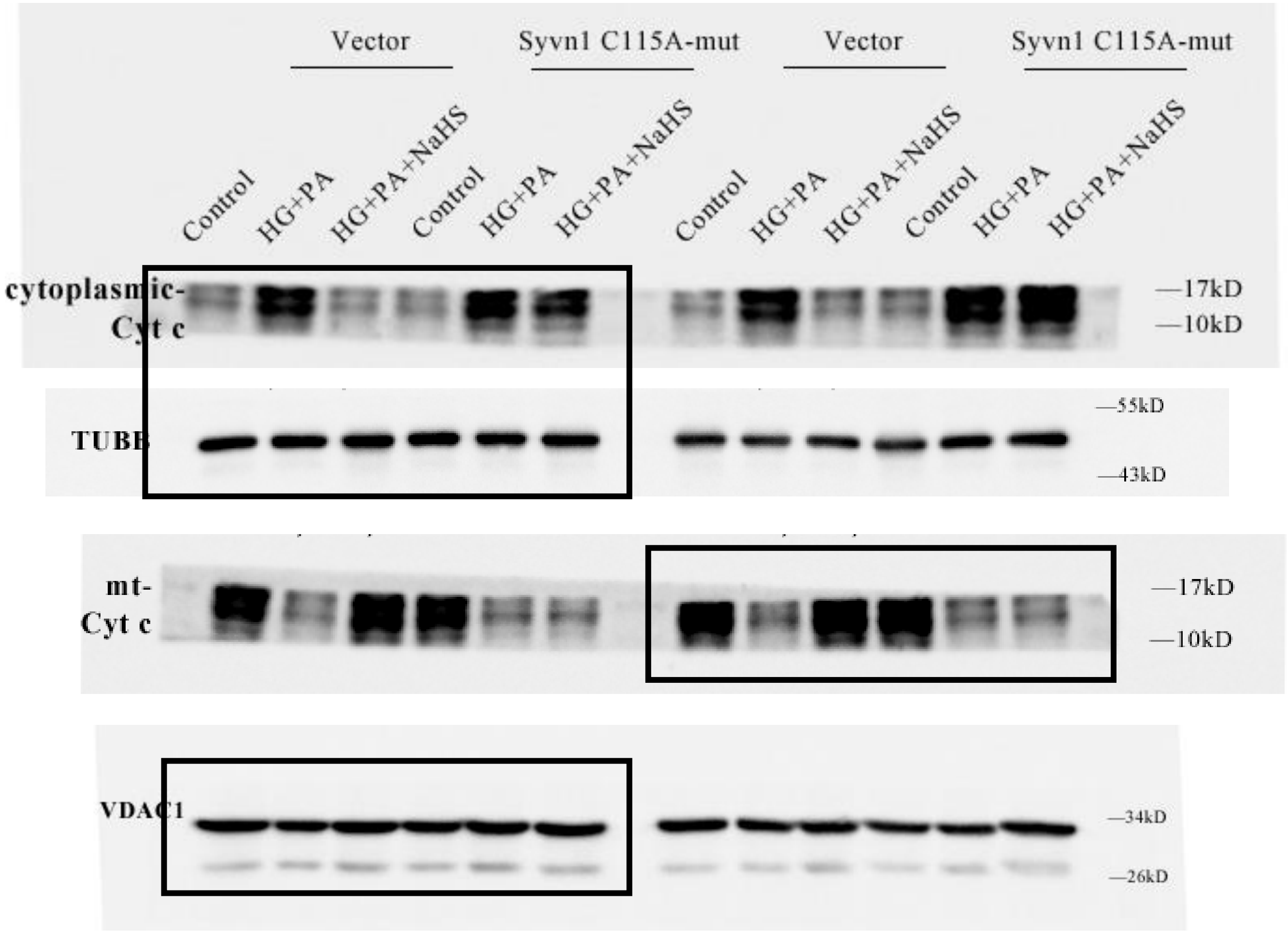

Full unedited gel for Figure 8D

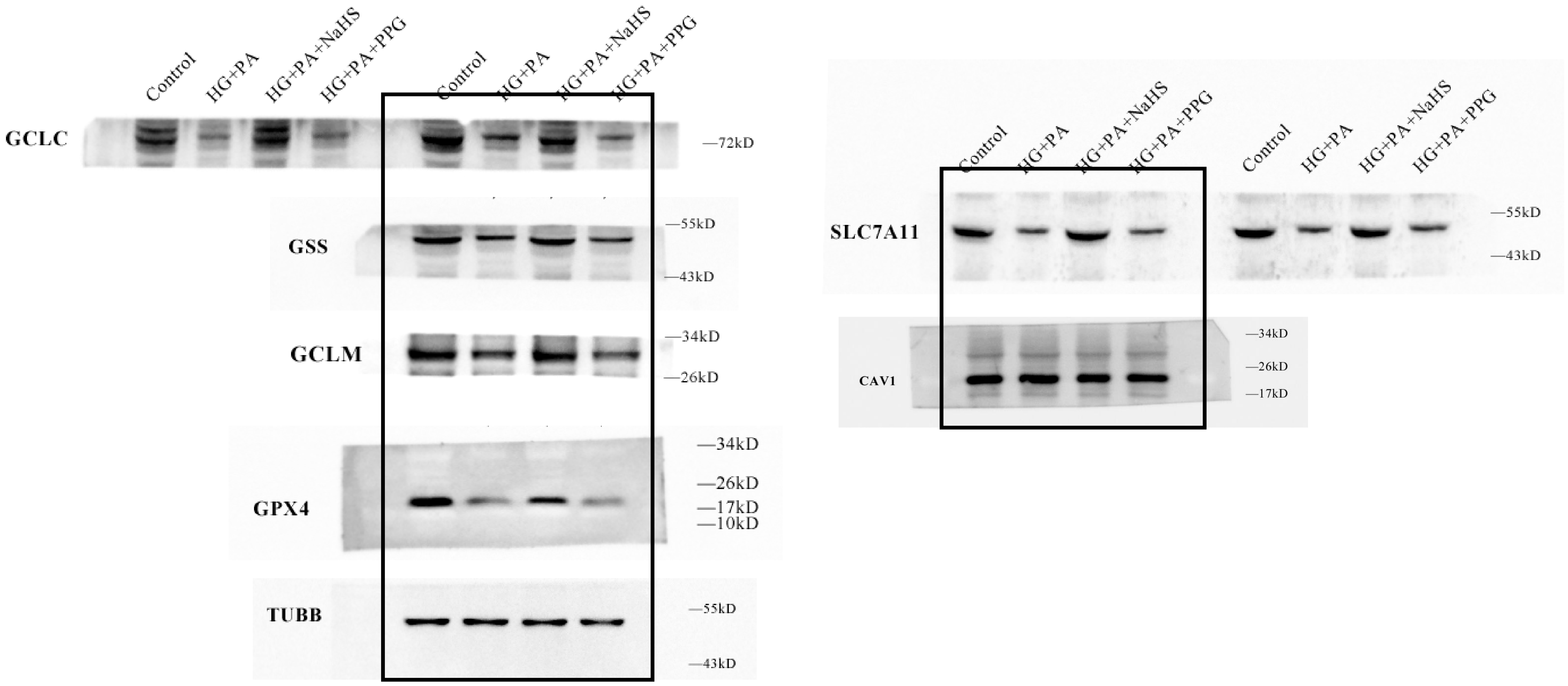

Full unedited gel for Supplemental Figure 2B

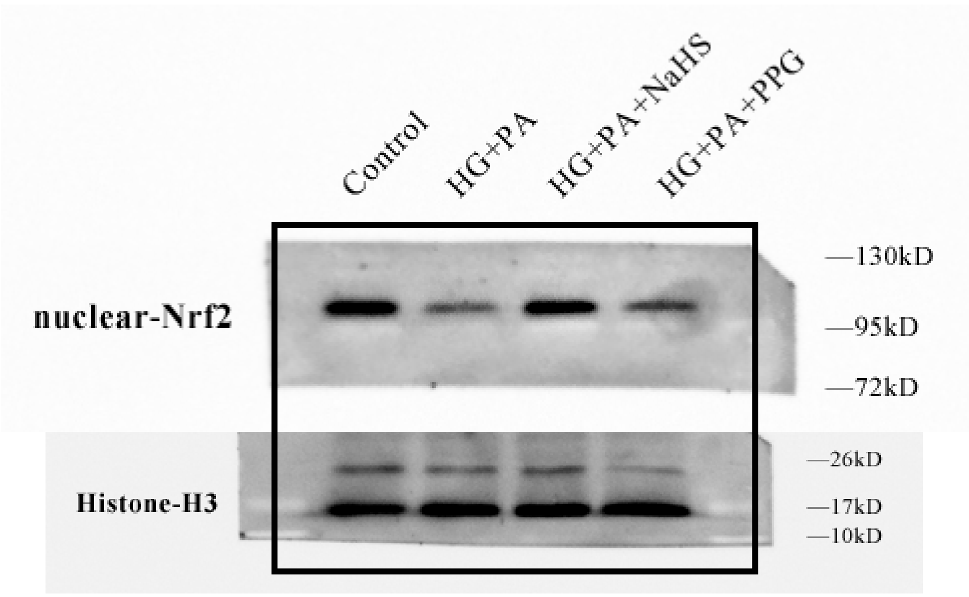

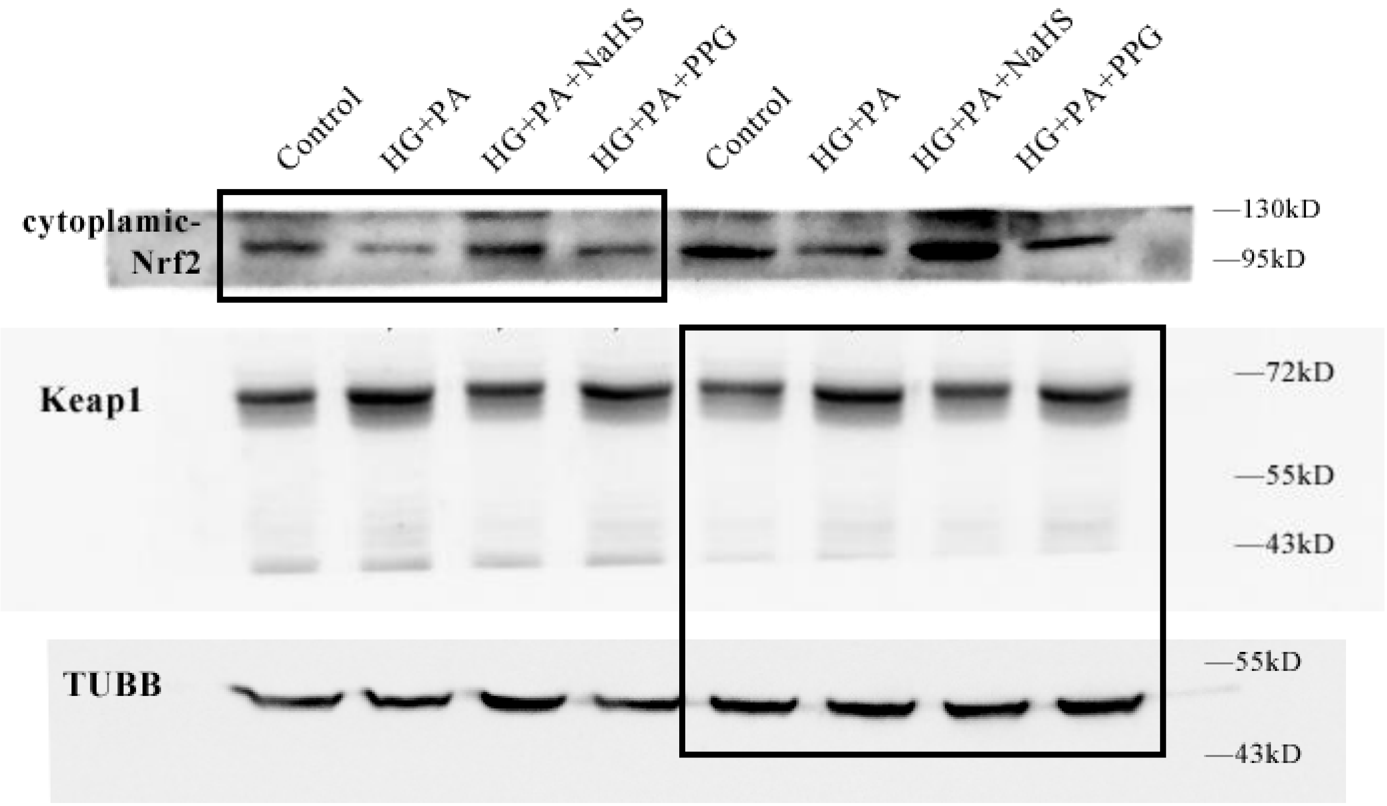

Full unedited gel for Supplemental Figure 4A

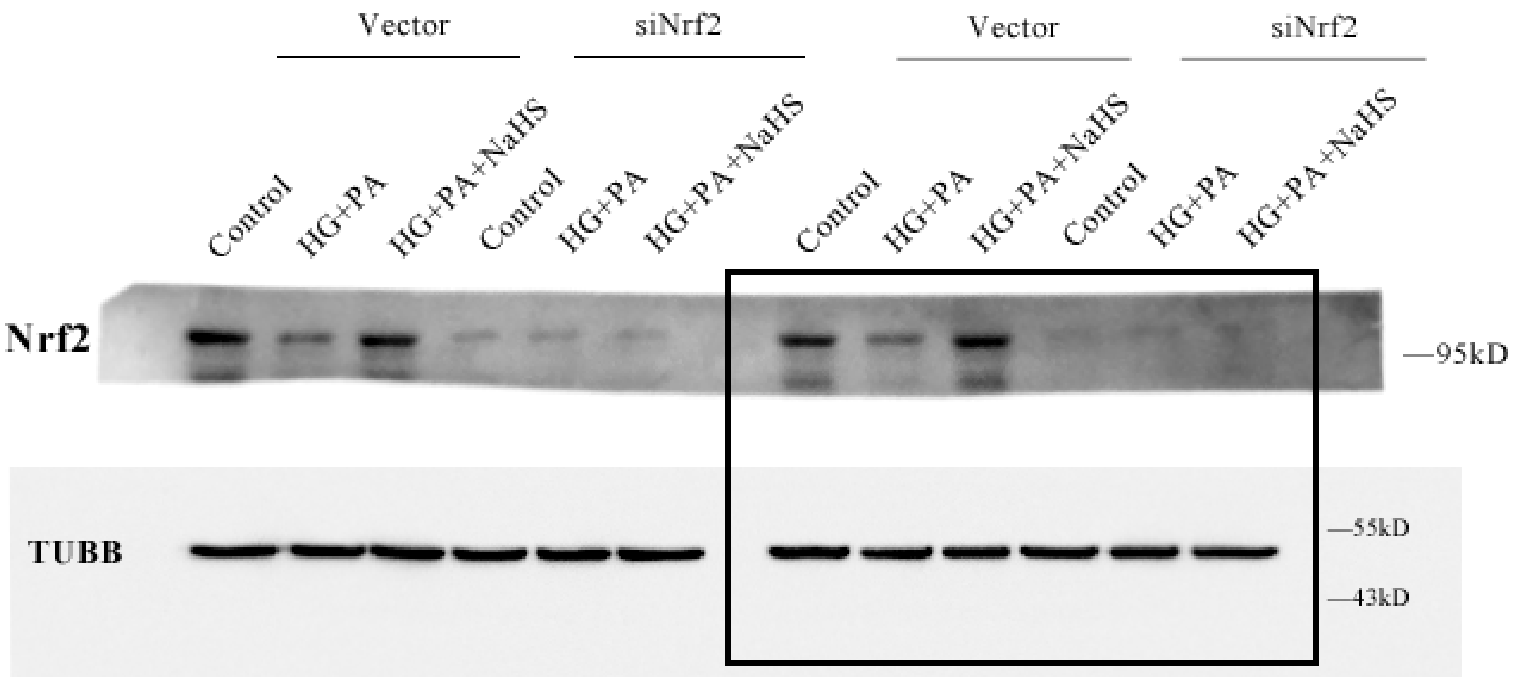

Full unedited gel for Supplemental Figure 4B

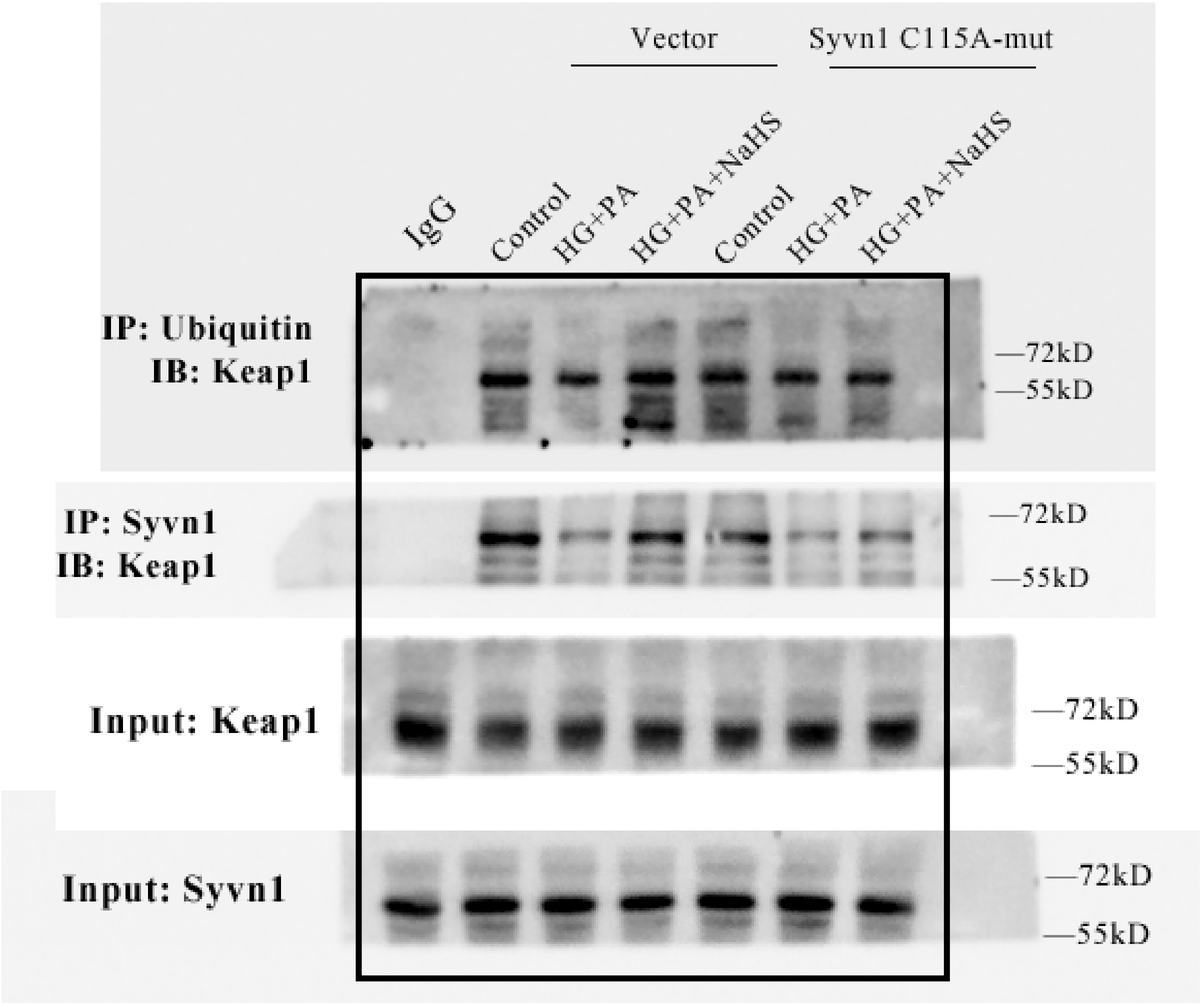

Full unedited gel for Supplemental Figure 5B
